# Temporally Coherent Backmapping of Molecular Trajectories From Coarse-Grained to Atomistic Resolution

**DOI:** 10.1021/acs.jpca.2c07716

**Published:** 2022-11-23

**Authors:** Kirill Shmilovich, Marc Stieffenhofer, Nicholas E. Charron, Moritz Hoffmann

**Affiliations:** † Pritzker School of Molecular Engineering, University of Chicago, Chicago, Illinois60637, United States; ‡ Max Planck Institute for Polymer Research, Mainz55128, Germany; § Weiss School of Natural Sciences, Department of Physics and Astronomy, Rice University, Houston, Texas77005, United States; ∥ Fachbereich Mathematik und Informatik, Freie Universität Berlin, Berlin14195, Germany; ⊥ Department of Physics, Freie Universität Berlin, Berlin14195, Germany

## Abstract

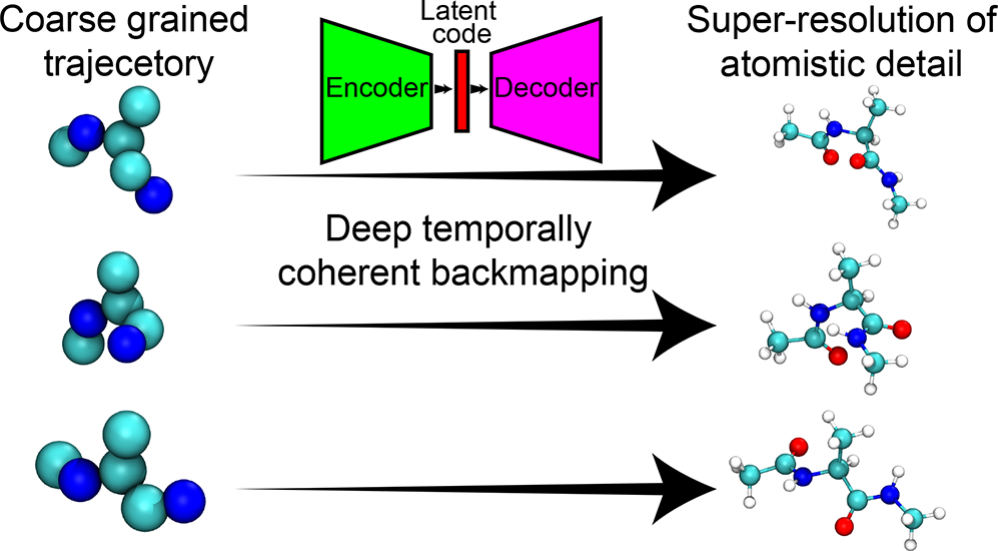

Coarse-graining offers a means to extend the achievable time and length scales of molecular dynamics simulations beyond what is practically possible in the atomistic regime. Sampling molecular configurations of interest can be done efficiently using coarse-grained simulations, from which meaningful physicochemical information can be inferred if the corresponding all-atom configurations are reconstructed. However, this procedure of backmapping to reintroduce the lost atomistic detail into coarse-grain structures has proven a challenging task due to the many feasible atomistic configurations that can be associated with one coarse-grain structure. Existing backmapping methods are strictly frame-based, relying on either heuristics to replace coarse-grain particles with atomic fragments and subsequent relaxation or parametrized models to propose atomic coordinates separately and independently for each coarse-grain structure. These approaches neglect information from previous trajectory frames that is critical to ensuring temporal coherence of the backmapped trajectory, while also offering information potentially helpful to producing higher-fidelity atomic reconstructions. In this work, we present a deep learning-enabled data-driven approach for temporally coherent backmapping that explicitly incorporates information from preceding trajectory structures. Our method trains a conditional variational autoencoder to nondeterministically reconstruct atomistic detail conditioned on both the target coarse-grain configuration and the previously reconstructed atomistic configuration. We demonstrate our backmapping approach on two exemplar biomolecular systems: alanine dipeptide and the miniprotein chignolin. We show that our backmapped trajectories accurately recover the structural, thermodynamic, and kinetic properties of the atomistic trajectory data.

## 1 Introduction

A central limitation of modeling soft-matter systems with molecular dynamics (MD) simulations is the long characteristic time scales of interesting processes, such as protein folding, compared to the relatively short integration time steps required to accurately propagate the system forward in time. A plethora of strategies strive to overcome this time scale barrier, such as enhanced sampling techniques,^1−5^ modern/specialized hardware,^6−8^ and hierarchical multiscale modeling.^9−14^ Coarse-grained (CG) simulations are one such multiscale approach that enables access to spatiotemporal scales entirely out of reach of conventional atomistic molecular dynamics simulations. The process of coarse-graining typically aggregates groups of atoms into “beads” or “superatoms” intended to preserve important properties of the original atomistic system.^15−17^ As such, CG simulations require monitoring fewer particles, which allows for the study of larger and more complex systems typically untenable in the atomistic regime at comparable computational cost. A typical consequence of this reduction in resolution is an effective “smoothing” of the underlying free energy surface, which helps expedite large-scale and slowly evolving conformational motions that might otherwise be frustrated or kinetically trapped in the more rugged atomistic landscape^12,18−21^ These advantages have led to the growing popularization and use of CG models, particularly for simulations of proteins, polymers, molecular self-assembly, membranes, and high-throughput screening.^22−31^

The primary concession of coarse-graining is the sacrifice of fine-grained, atomistic detail. Restoring this lost detail by converting a CG representation into a corresponding atomistic representation is commonly dubbed “backmapping” and is important for analyses requiring atomistic resolution, for example, electronic structure calculations for determining NMR spectra or dipole moments.^32,33^ Traditional backmapping strategies rely on geometric heuristics to replace beads with their associated atomic fragments. These approaches typically produce quite poor initial structures that must be subsequently subjected to refinement using energy minimization and/or (restrained) molecular dynamics to equilibrate each backmapped frame.^11,34−37^ However, significant computational cost is incurred with the required frame-by-frame intervention in these approaches, which hinders the applicability of backmapping larger systems and/or longer trajectories. More recently, data-driven backmapping techniques have been proposed which deploy machine learning (ML) models that learn to reconstruct atomistic details from training examples.^38−43^ These approaches offer more scalability with higher throughput as they are typically trained to produce well-equilibrated structures that do not require frame-by-frame energy minimization or relaxation. Coarse-graining is an inherently many-to-one operation, with multiple atomic structures corresponding to each CG representation. A favorable feature of any backmapping procedure is the capacity to recapitulate the conformational diversity of atomic structures corresponding to a particular CG representation. A subset of these data-driven methods^38,39,41,43^ that possess this conformational expressibility are therefore capable of nondeterministic backmapping, where a variety of feasible and novel atomistic structures can be generated when backmapping any individual CG configuration.

A commonality between all existing approaches is backmapping each frame individually and separately from one another. However, leveraging temporal information can improve reconstruction quality and enable the recovery of dynamic properties. In particular, some important dynamic properties rely on time correlations of local atomistic details. For example, the calculation of diffusion constants is related to the integral of the velocity autocorrelation;^44^ infrared absorption spectra are related to the autocorrelation function of the total dipole moment,^45,46^ and scattering functions are related to Fourier transforms of the van Hove correlation function.^47−50^ Existing backmapping schemes are not temporally aware, and correlations between consecutive frames are only maintained via large-scale characteristics. As a consequence, the reintroduced degrees of freedom between consecutive frames might decorrelate locally, and time correlations based on local, atomistic descriptors are typically not reliable for such backmapped trajectories. Therefore, presently absent from this suite of backmapping methods is a data-driven approach for generatively backmapping CG trajectories that also incorporates temporal information.

We present in this work a new method to perform temporally coherent backmapping of molecular simulation trajectories via a deep learning-based solution for all-atom reconstruction of CG simulation trajectories that aims at both generating well-equilibrated molecular structures for each frame and achieving temporal coherence between frames. To this end, we explicitly incorporate configurational information from previous simulation frames when generating reconstructions. This task is accomplished by training a conditional variational autoencoder (cVAE) that learns to up-scale CG configurations into full atomistic resolution by conditioning on the current coarse- and previous fine-grained structures. Our cVAE learns to model a variety of feasible atomistic structures associated with each CG configuration, which allows us to generatively produce novel backmapped trajectories that are not a simple carbon copy of the training data. We show for two exemplar biomolecular systems alanine dipeptide (ADP) and the miniprotein chignolin (CLN) that our approach generates atomic reconstructions which recover atomistic structural, thermodynamic, and kinetic properties. Furthermore, we show that our backmapping model performs well on held-out in-distribution data and generalizes to CG data originating from unseen and approximate CG force fields.

## 2 Methods

**Figure 1:**
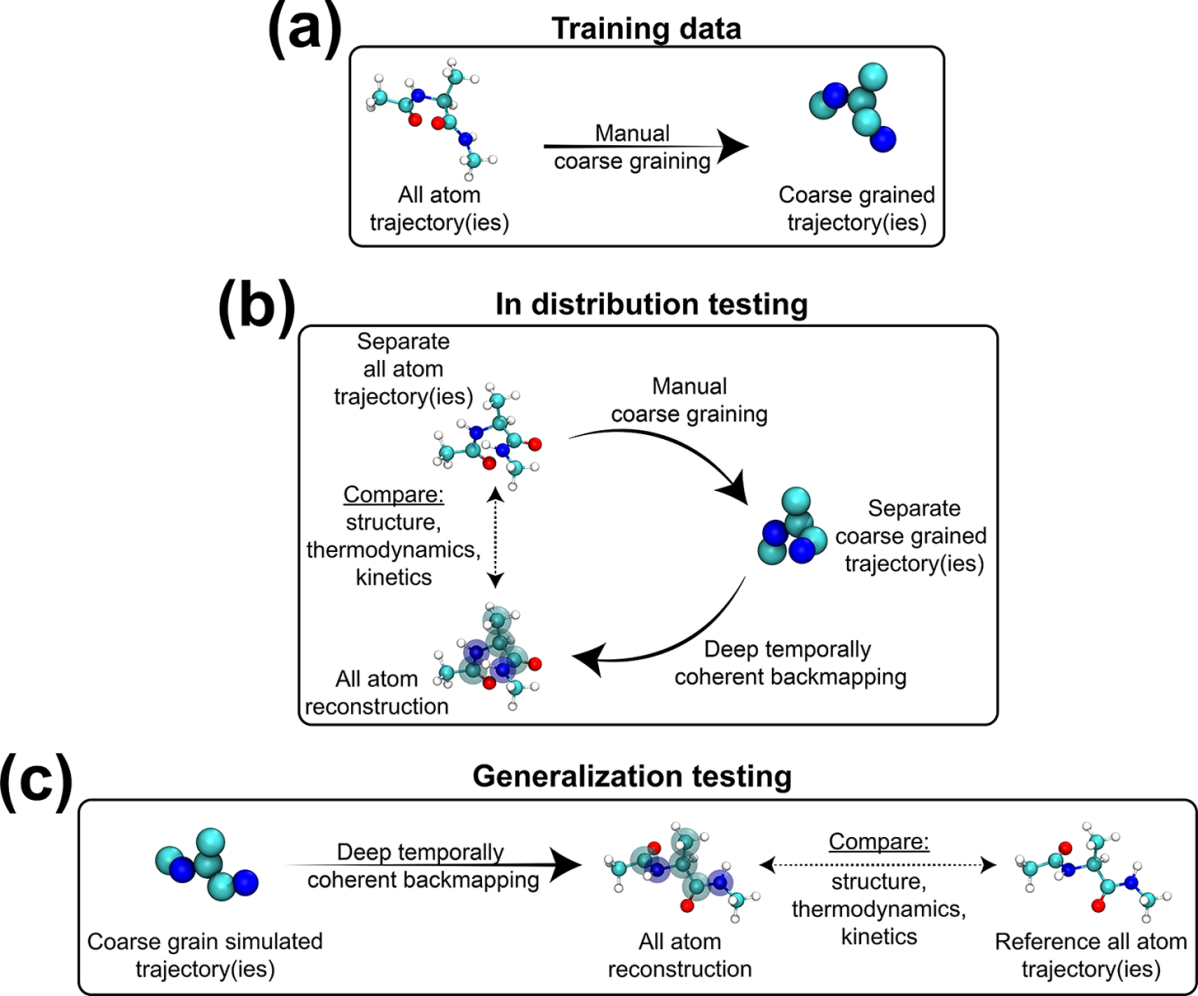
Illustration of training and testing setups.

Our approach utilizes a reference atomistic trajectory (or set of atomistic trajectories) for a molecule we intend to backmap. The trajectory contains *N* atoms and is composed of a collection of *T* frames AA = {AA^0^, AA^1^, ..., AA^*T*–1^}, where  are the atomistic coordinates for each frame. We assume there exists a coarse-graining function *f*_cg_ that maps all-atom coordinates to CG coordinates, such that , where *n* is the number CG beads such that *n* < *N*. This function is then applied frame-by-frame to the atomistic frames AA, yielding the corresponding CG trajectory CG = {CG^0^, CG^1^, ..., CG^*T*–1^}. This pair of atomistic and CG trajectories (AA, CG) composes the data used to train our super-resolution model (Figure 1a). From these data, our model attempts to learn the conditional distribution *P*(AA^*t*^|CG^*t*^, AA^*t*–1^) implicitly, such that we can reconstruct an atomisitic configuration given the CG structure CG^*t*^ and a previous atomistic structure AA^*t*–1^. Each training sample therefore consists of a sequential pair of atomistic configurations and the current CG configuration (AA^*t*^, CG^*t*^, AA^*t*–1^). While increasing the number of previous frames to incorporate AA^*t*–2^, AA^*t*–3^, etc. is straightforward, our experiments show that this simpler Markovian posture already yields accurate temporally coherent reconstructions that reproduce structural, thermodynamic, and kinetic properties with remarkable accuracy. A complete PyTorch^51^ implementation of our model with all associated analyses is publically available at DOI: 10.18126/tf0h-w0jz.^52^

### 2.1 Representing Molecules As Spatially Voxelized Particle Densities

Learning complex and high-order dependencies is a hallmark of computer vision. One of the most successful generative models is convolutional neural networks (CNNs), which have led to groundbreaking successes in image processing.^53−57^ In order to take advantage of CNNs for our backmapping task, we choose to represent our data as a set of 3D featurized images. To this end, a smooth density representation discretized on a 3D grid is used to encode the positions of atoms and beads. Each particle is placed in a separate tensor channel to avoid overlap of densities that would be difficult to disentangle. More concretely, an atomistic configuration  is represented as a 4D tensor , where the first three dimensions discretize the location of each particle in space and the final dimension represents a channel for each particle:
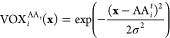
1Here, *i* is the particle index and  is the Cartesian location of each grid point taken from a regular Cartesian grid of width *r*_grid_ such that the maximal and minimal values of each spatial dimension are  and . The density of each particle is therefore a 3D Gaussian centered about the position  with a width of σ. It is critical to ensure that *r*_grid_ is large enough such that each particle density within the trajectory is fully enclosed by the voxelized grid. The parameter *d* then controls the resolution of our spatial discretization, such that a larger *d* leads to more spatial resolution at the cost of higher memory and processing requirements. Furthermore, σ, which controls the effective size of each particle density, will also impact the mass assigned to each voxel.

From a density profile  we can also recover a set of particle coordinates by performing a weighted average over the voxelized grid
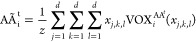
2where  is a normalization constant, *x*_*j*,*k*,*l*_ is a particular coordinate value within the three-dimensional spatially voxelized grid, and the summations are carried over the three spatial dimensions. Given a predicted density profile  we can perform this weighted average for each *i* particle channel  to recover the complete set of atomistic coordinates . We note that transforming these densities into and from Cartesian coordinates constitutes a sequence of differentiable operations and therefore enables us to readily incorporate this particle averaging and voxelization within the computational graph of our model. CG configurations CG^*t*^ are treated in the same way as their atomistic counterparts, yielding corresponding voxelized representations  where *n* is the number of beads associated with each CG configuration.

### 2.2 Conditional Variational Autoencoder

**Figure 2:**
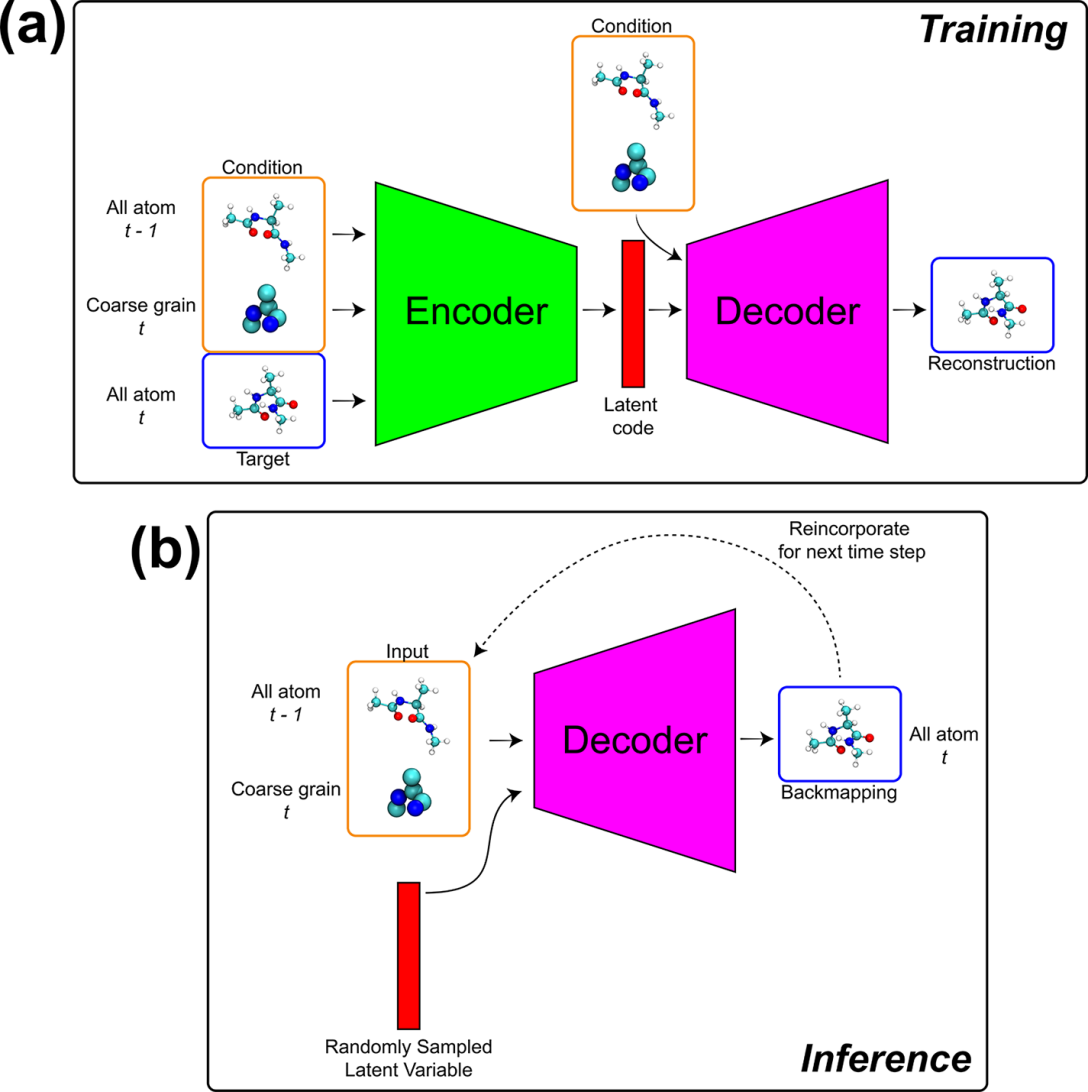
Schematic illustration of our model training and inference setups.

To learn a temporally coherent probabilistic mapping from CG to atomistic configurations, we train a conditional variational autoencoder (cVAE).^58^ A typical VAE^59^ consists of an encoder that compresses high-dimensional inputs into a lower dimensional latent space that captures salient information characterizing the input data. This latent, compressed representation is then inputted to a decoder that aims to reconstruct the original high-dimensional input. The cVAE operates under a similar premise, but the decoder is also provided with some partial information about the input along with the latent code when producing reconstructions. Figure 2a shows a schematic illustration of our cVAE architecture. In our application, the cVAE encoder takes as input the triplet of configurations (AA^*t*^, CG^*t*^, AA^*t*–1^), where the subset (CG^*t*^, AA^*t*–1^) is interpreted as the conditional variable and AA^*t*^, the intended reconstruction target. The decoder is a function of both the latent code and the conditional variable (CG^*t*^, AA^*t*–1^) and learns to reconstruct atomistic configurations  to closely match the data AA^*t*^. For a fixed condition (CG^*t*^, AA^*t*–1^), we can learn to meaningfully encode information about the target configuration AA^*t*^ into a low dimensional latent code, such that the decoder when presented with (CG^*t*^, AA^*t*–1^) yields slightly different, yet valid, reconstructions  for different instantiations of the latent code. This data driven approach enables our model to generatively produce reconstructions AA^*t*^ in a temporally aware way by conditioning on both the coarse grained configuration and the previous atomistic configuration (CG^*t*^, AA^*t*–1^) (Figure 2b).

#### 2.2.1 The Encoder

The purpose of the encoder in our cVAE is to distill information from the input configurations (AA^*t*^, CG^*t*^, AA^*t*–1^) into a low-dimensional latent space vector that is used in conjunction with the conditional variables (CG^*t*^, AA^*t*–1^) for the decoder to generate an atomistic reconstruction . These configurations are passed to the encoder as voxelized grids of particle densities  which are constructed as a single monolithic tensor concatenated along the particle dimension , where *N* and *n* are the number of atoms/beads in each atomistic and CG configuration, respectively. The encoder is composed of 3D residual CNNs with a terminal dense layer to extract a fixed dimensional latent vector. The locality of the CNN kernel provides a strong inductive bias by focusing on proximate spatial profiles from multiple high and low resolution scales and multiple time steps. Processing the input **x** using many consecutive CNN modules enables us to hierarchically incorporate progressively more distant features, and ultimately yielding a multiscale, spatially and temporally aware latent representation **z** ∈ *d*_latent_ for that slice of the trajectory. This latent representation **z** is then passed to the decoder in conjunction with the conditional variable  to predict the all atom configuration for the subsequent time step .

#### 2.2.2 The Decoder

The purpose of the decoder is to use information provided in the conditional variable  jointly with the latent code **z** produced by the encoder to reconstruct the current atomistic configuration AA^*t*^. Upon completing training, we can eschew the encoder and use the decoder as our generative model for backmapping. Using a randomly sampled latent variable **z** as a source of noise while specifying the conditional variable as the current CG configuration CG^*t*^ and the previous atomistic configuration AA^*t*–1^, we can backmap the atomistic reconstruction . Similar to the encoder, our decoder is primarily composed of residual 3D CNNs. Unlike the encoder, the neural network backbone of the decoder needs to only output a 4D tensor of equivalent dimensionality to the voxelized atomistic representation . As the voxelized representation  contains separate channels for each atom, we can simply perform an average over the spatial density profiles within each particle channel to independently localize each atom coordinate (c.f. section 2.1). We control the behavior of our model by defining a training loss that captures relevant aspects we ultimately want reflected in our reconstructions.

### 2.3 Training Routines

Our model is trained end-to-end using the ADAM optimizer.^60^ For a single sample, the complete loss  is given by
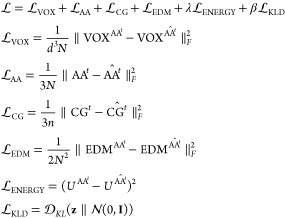
3

The first five terms in eq 3 are components of an effective reconstruction loss, and  is the standard Kullback–Leibler divergence loss between the latent code **z** and a normal distribution typically used in VAE training.^59^ While we ultimately strive to recover all atom coordinates AA^*t*^, the model primarily operates on spatially voxelized particle density representations . A critical component of learning will therefore involve reconstructing these density profiles to enable accurate and sharp atomic coordinate generation. The  term is a mean squared error (MSE) between the target  and reconstructed  atomistic voxels, while  is an MSE between the ground truth AA^*t*^ and reconstructed  atomic coordinates.  helps the model learn to reproduce the atomic densities of the intermediate voxelized representations, while  helps ensure sharper coordinate reconstruction when the voxels  are ultimately collapsed back into atomic coordinates AA^*t*^. We also use the coarse graining function *f*_cg_ to determine the CG representation of an atomistic reconstruction , which is used in  to calculate an MSE with respect to the input CG coordinates CG^*t*^. The motivation to include  is that a coarse graining of the atomistic backmapping  should ultimately match the original CG structure CG^*t*^ from which the reconstruction is derived. In the  term, we calculate an MSE between the *N* × *N*-dimensional Euclidean Distance Matrix (EDM) of the target  and the reconstructed  atomic coordinates to help our backmapping better preserve bond lengths and other interatomic distances. Last, in the  term, we calculate an MSE between the scalar valued total potential energy of the target  and reconstruction .  serves as a regularizer to improve the quality of backmapped structures by penalizing reconstructed configurations that may have suitable geometric contributions but are otherwise energetically unfavorable. As such, it accelerates convergence and helps more precisely match the reconstructed energetics to the ground truth trajectory. During training, before configurations are voxelized, each training configuration is mean centered, and we learn covariance with respect to rigid rotations by applying a random Euler rotation  separately augmenting samples in each forward pass (AA^*t*^, CG^*t*^, AA^*t*–1^) → (AA^*t*^**R**, CG^*t*^**R**, AA^*t*–1^**R**).

A challenge of incorporating  within the loss function is that the potential energy function *U* is sensitive to small perturbations of the atomic coordinates, which is most severe for the bonded and nonbonded Lennard-Jones interaction. As such, it can become dominatingly large during the early stages of training before the model learns to stably localize atomic coordinates. To alleviate this issue, we incorporate the prefactor λ in eq 3, which we set to λ = 0 for a fixed number of initial training steps, after which point λ is slowly annealed up to λ = 1 using an exponential annealing schedule. We also include the β prefactor alongside  for more flexibility in balancing the impact of the KL regularization against the reconstruction losses. For the ADP model, we employ a cyclic annealing schedule for β to mitigate KL vanishing,^61^ while for the CLN model we simply maintain β = 1 throughout training. Complete training settings and hyperparameter details are presented in the Supporting Information.

### 2.4 Inference

At inference time, we omit the encoder and use the decoder as the primary tool for generatively backmapping a CG trajectory. The decoder thereby reconstructs the atomistic frames in an autoregressive manner, i.e., the previous reconstructed atomistic frame  serves as the input for the reconstruction of the next frame . More specifically, the input for the decoder consists of a fixed dimensional latent vector **z** and the conditional variable  (Figure 2b). However, for the first trajectory frame *t* = 0, there is no preceding frame for us to ascertain AA^–1^. In this case, an atomistic configuration from the training data set is chosen as this initial seed configuration AA^–1^. We select this configuration by first determining the trajectory frame t* in the training data set AA_train_ that minimizes the RMSD with respect to the first test set CG frame , and then simply set the initial seed configuration as the immediately preceding training set trajectory frame . Last, apply a rigid rotation to align AA^–1^ with CG^0^.

At inference time, the decoder uses a fixed dimensional latent vector **z** to provide a source of variance when generating atomic reconstructions. For a fixed condition **c**, our model learns to effectively produce valid yet slightly different atomic reconstructions  for different instantiations of the latent code **z**. Typically, inference is performed for VAEs or cVAEs by simply sampling **z** for each inference pass from the prior distribution over **z**, which is normally taken as an isotropic Gaussian .^59^ At training time, however, the decoder is exposed to latent codes produced by the encoder from only the training set **Z** = {**z** = encoder(**x**): **x** ∈ **X**_train_}. Therefore, any mismatch between the true aggregated posterior **Z** and the assumed prior *p*(**z**) can lead to poor generative performance by the decoder when samples are selected from *p*(**z**) because this may lead to operating within regions of latent space previously unseen by the decoder during training. We remedy this issue, following ref (62), by performing an ex-post density estimation fitting a 10-component Gaussian Mixture Model (GMM) over the true posterior **Z**. We then at inference time randomly sample **z** from our fit GMM, instead of from the assumed prior . This process ensures that our decoder operates within densely sampled latent space regions, which leads to higher fidelity, and less error-prone, atomic reconstructions.

After completing training for both our ADP and CLN models, we can visualize our latent space posterior **Z** by projecting our full-dimensional latent space into the first two Principal Components (PCs) of **Z** (Figure S5 in the Supporting Information). Color-coding latent codes by the potential energy of the corresponding target configuration *U*_target_ in each sample reveals a strong correlation between the leading PC and internal energy for both of our test systems ADP  and CLN . These strong correlations suggest that our encoder effectively extracts features from the input data to encode physically meaningful features, reflected in the internal energy, of the target atomistic configuration within our latent space embedding. We can also interrogate the generative capabilities of our model by using our decoder to produce reconstructions of a fixed CG input under many different latent code instantiations (Figure S6 in the Supporting Information). The diversity of valid generated structures which still adhere to the CG input suggests that the decoder is attentive to subtle variations in the latent code when reconstructing atomistic configurations. In all, the properties of our cVAE model and latent space embedding validate expected behavior: our encoder effectively distills information to the latent code from the input data, while our decoder purposes the latent code together with the condition to nondeterministically and generatively reconstruct atomistic structures.

### 2.5 Data Curation

Our model learns to backmap a CG trajectory of a molecule by training on a reference atomistic trajectory of that molecule that we coarse-grain after the fact to yield exemplar pairs of atomistic and coarse grained frames (Figure 1a). When backmapping to predict an atomistic structure AA^*t*^, we consider both the current CG structure CG^*t*^ along with the previous atomistic structure AA^*t*–1^. An important consideration when obtaining training data is the temporal spacing between consecutive frames AA^*t*–1^ and AA^*t*^, as it may be impossible to accurately recover molecular motions that occur faster than this time. We also find that it is critical to ensure that the reference atomistic trajectory is sufficiently sampled and captures the relevant atomistic state transitions that are expected to be reflected in the backmapped atomistic trajectory. These aspects of appropriate conformational sampling are important considerations in our approach and more broadly in data-driven backmapping schemes, to provide confidence when backmapping data originating from coarse-grained models as they are typically designed to improve sampling of rarely occurring atomic conformational states. For example, a data-driven backmapping model strictly trained on simulation data from a protein simulation in the folded ensemble is unlikely to reliably and effectively backmap coarse-grained configurations in the unfolded ensemble. While ensuring comprehensive coverage of the accessible conformation space in molecular simulations remains an open challenge, a continually growing library of enhanced and adaptive sampling techniques,^63−70^ along with other classical techniques such as simulated annealing, can serve to provide landmark atomistic configurations to initialize unbiased simulations in the collection of a diverse and through training data set.

We train separate models on reference atomistic trajectories of alanine dipeptide (ADP) and the mini protein chignolin (CLN). Separate held-out trajectories for ADP and CLN are used as test sets for evaluating the performance of our model in-distribution data (Figure 1b). As a more challenging generalization test of our method, we also backmap CG trajectories generated from a bespoke CG force-field, CGSchNet (Figure 1c). We then evaluate structural, thermodynamic, and kinetic statistics of our atomistic reconstructions against reference atomistic trajectories to evaluate the performance of our method. In the following sections, we describe the details of the simulation methods used to generate the ADP and CLN trajectories we use to train and test our model.

#### 2.5.1 Alanine Dipeptide

##### Atomistic Data

Atomistic trajectories of alanine dipeptide (ADP) used for training are collected by performing molecular dynamics simulations in explicit solvent using OpenMM.^71^ We closely mimic the simulation procedures outlined in ref (72). Langevin dynamics simulations are performed with a 2 fs time-step in the NVE ensemble using the AMBER ff-99SB-ILDN force field^73^ within a cubic box containing 651 TIP3P water molecules randomly placed within a volume of 2.7273 nm^3^. Electrostatics are treated using the particle-mesh Ewald (PME) method^74^ using a 1.0 nm cutoff for the direct space interactions. The lengths of all bonds involving hydrogen atoms are constrained. Steepest descent energy minimization is used to relax the initial system configuration to within an energy of 10 kJ/mol. We then assign initial velocities to the energy minimized configuration by sampling from a Maxwell–Boltzmann distribution at 300 K. A short 100 ps equilibration run is then performed, followed by a 500 ns production run. This 500 ns production run comprises our training data. A separate 250 ns production run is performed to generate the in-distribution data used for testing. In each case, trajectory snapshots are saved every 1 ps, yielding 500 000 simulation frames for the training data and 250 000 simulation frames for the test data.

##### Coarse-Grained Data

For the CG representation of ADP, we choose to remove all solvent and represent the molecule using the five backbone carbon and nitrogen atoms (C, N, CA, C, N) and the carbon beta (CB) of the alanine residue, resulting in a total of six coarse grain atoms. A CG trajectory is generated from the above-mentioned all atom trajectory by slicing the coordinates to retain only the specified coarse grain atoms. The same coarse grain mapping is also applied to the all-atom forces to produce an associated set of instantaneous coarse grain forces. Using both the coarse grain coordinates and forces, bespoke CG force fields are recovered using CGSchNet^75^ neural network models. These ADP CG models are trained using the same data set from refs (75) and (76) and are then used to generate out-of-sample data in the form of CG trajectories as a generalization test for our backmapping method to illustrate performance on real, noisy data. The trajectories used for backmapping consist of 100 separate simulations initialized at random configurations from the reference atomistic data set and containing a total of 4000 frames each. Sequential CG simulation frames are temporally separated by the same 1 ps spacing as used in the training data trajectory. The training routines and hyperparameters of these CG force field models, as well as the simulation parameters for the out-of-sample CG simulations, are described in the Supporting Information.

#### 2.5.2 Chignolin

##### Atomistic Data

We use reference Chignolin (CLN) trajectories generated from atomistic simulations performed in ref (76). Briefly recapping the protocols, these simulations are performed using the ACEMD program^77^ on GPUgrid^78^ at 350 K mimicking the setup originally used on the Anton supercomputer simulation.^79^ To sufficiently sample folding/unfolding transitions in CLN, the data are produced through an MSM-based adaptive sampling strategy, which expedites conformation sampling for this system up to an order of magnitude,^80^ consisting of an aggregated ∼187 μs of molecular dynamics simulation split into 3744 short trajectories. Simulation snapshots are spaced by 100 ps culminating in a total of 1 868 861 frames. As each of the 3744 trajectories are independent we simply split-off 3650 for training and the remaining 94 for testing to comprise our in-distribution test set.

##### Coarse-Grained Data

For a CG representation of CLN, we choose to remove all solvent and represent the molecule using just the 10 sequential α-carbon (CA) atoms along the molecular backbone. Following the same procedure described above for ADP, a set of CG trajectories and associated forces are generated. The same CG coordinates and forces mapped from the atomistic data described in the preceding section are then used to train CGSchNet^75^ neural network force fields, which in turn are used to generate out-of-sample data for generalization tests of our backmapping model. We produce 1000 separate trajectories containing 4000 frames each and, similar to ADP, are initialized at random configurations from the reference atomistic data set. In this case, for CLN, each frame is temporally separated by 1 ps, which differs from the 100 ps frame spacing in the training data. While we appreciate that this frame spacing represents a different regime than our model is trained to operate in, the inherently accelerated nature of CG dynamics makes direct comparison between CG and atomistic time steps difficult. We appeal to a smaller frame spacing in this work to account for this inherent acceleration of CG dynamics and enable better sampling of short-lived, transient states within these trajectories. As with ADP, the training routines, model hyperparameters, and CG simulation parameters for the CLN coarse grain force fields are detailed in the Supporting Information.

## 3 Results

We present a data-driven and temporally coherent approach for backmapping coarse grained trajectories into full atomistic resolution. Our approach is based on training a conditional variational autoencoder (cVAE) to generate atomistic coordinates of a coarse grain (CG) configuration while also incorporating information from the previous atomistic configuration within the trajectory. The proposed method is applied to two biomolecular systems: alanine dipeptide (ADP) and the miniprotein Chignolin (CLN). The performance of our model is evaluated by measuring its ability to generate backmapped trajectories that preserve atomistic structural, thermodynamic, and kinetic properties. Our model is trained on data consisting of pairs of atomistic and corresponding CG trajectories, that are obtained by mapping the atomistic data to CG resolution (Figure 1a). For one evaluation test, we apply our method to backmap CG trajectories generated from mapping separate held-out atomistic trajectories to CG resolution, which will be referred to as the *in-distribution* test set, or simply referred to as the *test set* (Figure 1b). For a more challenging test of the generalization capabilities of our model, we apply our method to CG trajectories generated using a bespoke CG force-field CGSchNet,^75^ which will be referred to as *out-of-distribution* or *generalization* set (Figure 1c). For both molecules ADP and CLN, the in-distribution and generalization tests show excellent agreement in measures of structural, thermodynamic and kinetic similarity between our backmapped and atomistic trajectory data.

### 3.1 Alanine Dipeptide

#### 3.1.1 Energetics

**Figure 3:**
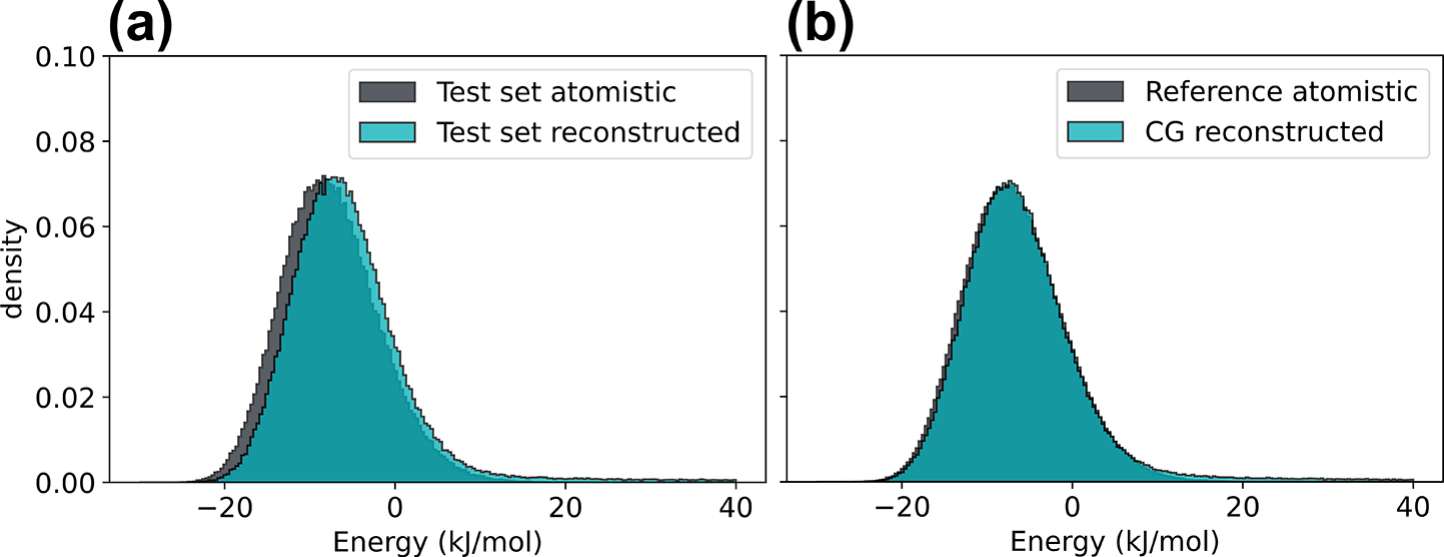
Comparison of the internal potential energy distributions for atomistic and backmapped ADP trajectories. (a) Internal energies for the held out test set trajectory and the atomistic reconstructed trajectory generated by backmapping a manual coarse graining of the original atomistic trajectory. (b) Generalization test comparing reference atomistic trajectories obtained from ref (81) to our model-generated backmappings of a real CG simulation conducted using CGSchNet.^75^

**Figure 4:**
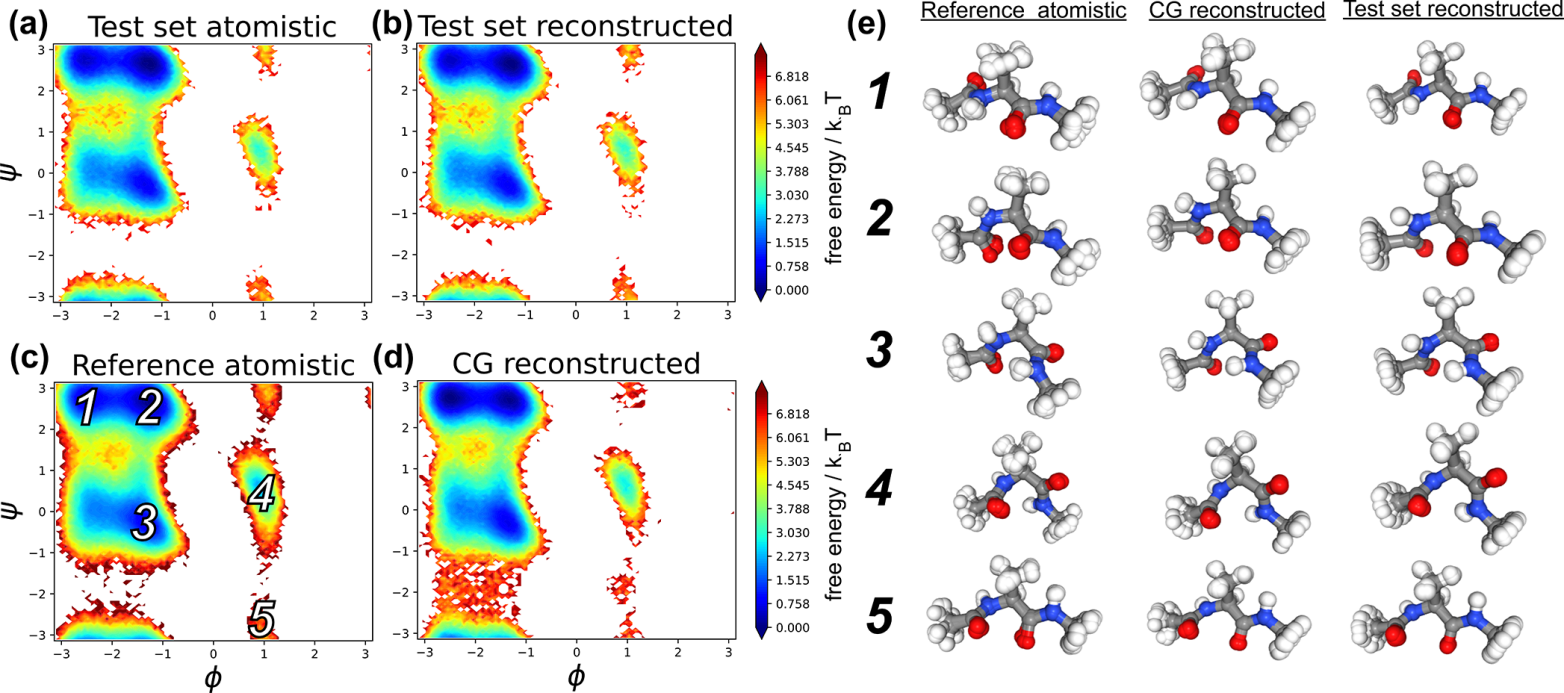
Comparison of atomistic and backmapped MSM-reweighted Free Energy Surfaces (FES) for ADP. Ramanchandran plots for the in-distribution test set that includes (a) a held-out atomistic trajectory and (b) our model generated backmapping of the manually coarse grained atomistic trajectory. For a more challenging test of generalizability, we compare the Ramachandran plots for (c) a reference atomistic trajectory taken from ref (81) and (d) a backmapping of a CG simulation performed using CGSchNet.^75^ Labeled in c are phase space locations for the five metastable states of ADP. (e) Seven superimposed configurations near each of these five major metastable states from (c) the reference atomistic, (d) the CG reconstructed, and (b) the test set reconstructed trajectories.

As a first example, we apply our backmapping method to the small molecule alanine dipeptide (ADP) composed of 22 atoms from which we consider a coarse-graining into six CG beads along the peptide backbone. We evaluate structural similarity between atomistic and our backmapped trajectories by comparing distributions of the internal potential energy. The internal energy aggregates contributions from bonded and nonbonded interactions. As such, agreement between atomistic and reconstructed energy distributions serves as a good indicator of overall structural similarity. The energy distribution for the in-distribution test set shown in Figure 3a reveals that our model nearly identically reproduces the energy distribution of the original atomistic data. Similarly, the energy distribution for the generalization test set obtained by backmapping a trajectory generated with CGSchNet^75^ is displayed in Figure 3b. This out-of-distribution test represents a more difficult exercise for our model, as it must generalize to real CG simulated data generated by a different, approximate force field that our model was not exposed to during training. Our model yields backmapped structures that nearly identically match the energetics of the atomistic reference data. The only noticeable deviations are minor shifts in the energy distributions, which are a consequence of a slightly increased population of high-energy backmapped reconstructions (Figure 3). While these rare high-energy configurations can be attributed to minor deviations in a few bond length and angle distributions, we find overall excellent agreement between distributions of local intramolecular features between backmapped and reference structures (Figures S7–S10 in the Supporting Information). Our backmapping method in general reconstructs atomistic ADP trajectories with high structural and energetic similarity to reference atomistic data while also generalizing well to unseen and real CG force fields and, as a result, generates visually identical backmaped structures (Figure 4e and Figure S6 in the Supporting Information).

#### 3.1.2 Thermodynamics

We evaluate thermodynamic similarity by comparing free energy surfaces (FES) of our reconstructed trajectories to reference atomistic data. We construct our FES in the space of the backbone dihedral angles (ϕ, ψ) as they have proven to be good collective variables for characterizing the conformational states of ADP.^82,83^ In Figure 4a, we present the FES in (ϕ, ψ) for the in-distribution test set atomistic trajectory compared to the CG backmapped trajectory FES in Figure 4b. Our atomistic reconstruction here reproduces a nearly identical FES to the ground truth atomisitic data, demonstrating that for in-distribution data our model accurately captures the ground truth thermodynamics. For the generalization test, we also present an FES generated from a reference atomistic trajectory (Figure 4c) compared to the FES of a CG backmapped trajectory generated from a CG simulation performed with CGSchNet^75^ (Figure 4d). Once again, our reconstruction is in excellent agreement with the atomistic reference, importantly correctly identifying the five major metastable states of ADP. In Figure 4e, we show a superimposed collection of configurations for each of these metastable states from the reference, test set reconstructed, and CG reconstructed trajectories. For both in-distribution and out-of-distribution data, our model reconstructs visually identical configurations with remarkable similarity to the atomistic reference data. Note that the CG model yields configurations throughout the transition paths between metastable states, for example, (ϕ ≈ −2, ψ ≈ −2). While those configurations are under-represented in the atomistic trajectory due to high-energy barriers, the smoothed energy landscape of the CG force field enables broader and more frequent exploration of these regions of phase space. We find our model generalizes well to those sparsely sampled areas and accordingly reconstructs these high-energy configurations (Figure S11 in the Supporting Information). Overall, our backmapping scheme reproduces the FES for ADP that is in excellent thermodynamic agreement with reference atomistic data for both our in-distribution and generalization tests.

#### 3.1.3 Kinetics

An important aspect of the proposed method is the incorporation of the previous trajectory configuration as a conditional input for our ML model. This provides temporal information required to achieve temporal coherence between consecutive frames, which is typically omitted in traditional backmapping strategies. Here, we test the temporal coherence of our backmapped trajectories by analyzing kinetics in terms of implied process time scales and velocity distributions.

**Figure 5:**
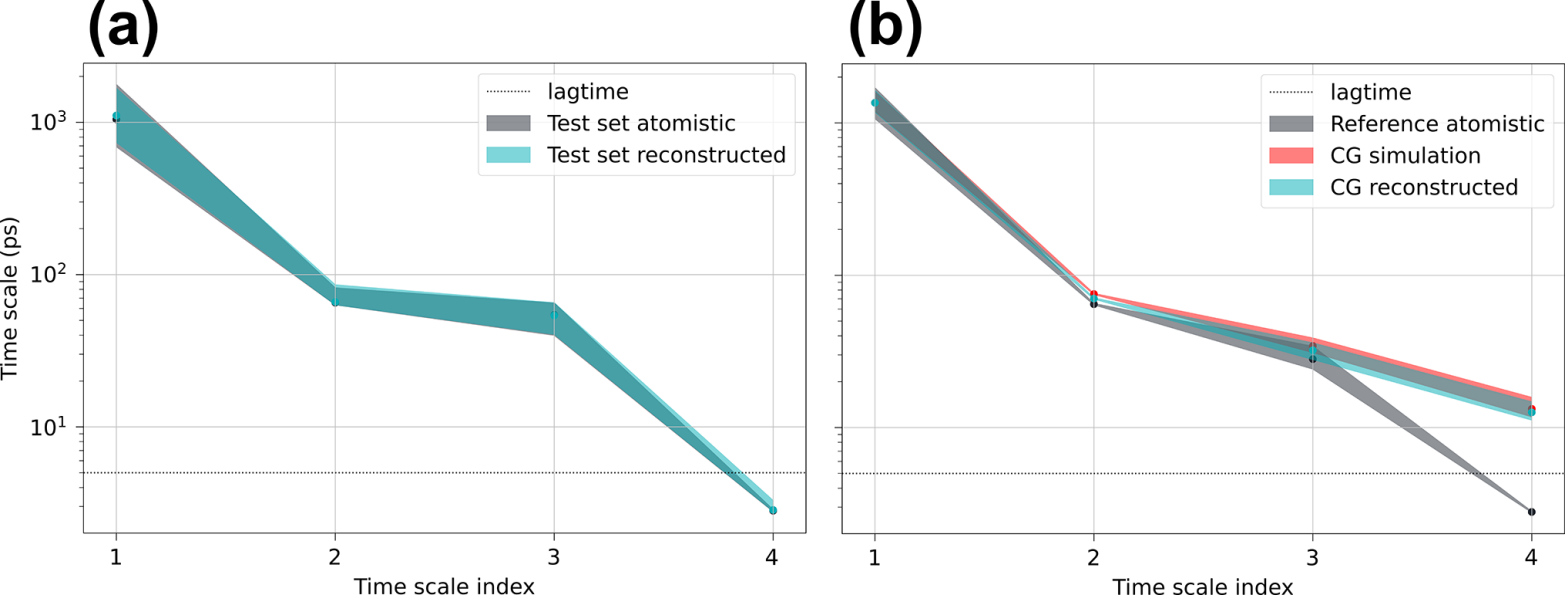
Implied time scales of atomistic and backmapped CG trajectories for ADP. (a) Comparisonon of time scales recovered for the test set atomistic and test set reconstructed trajectories. (b) Implied time scales calculated for the reference atomistic data taken from ref (81) compared to the backmapped CG simulation performed with CGSchNet^75^ along with the original CG simulation. Time scales for the backmapped and CG simulated data are normalized such that the dominant processes is the same as the reference atomistic trajectory. Errors are estimated with Bayesian sampling and represent a 95% confidence interval of the time scales of the sampled MSMs.

We compare kinetic agreement between ground truth atomistic data and our backmapped reconstructions by building Markov State Models (MSMs)^84−88^ to characterize and compare the recovered physical processes and time scales. Construction and estimation of MSMs are carried out using the Deeptime^88^ software library. We construct MSMs in ϕ,ψ space for ADP and perform state assignment by discretizing the phase space with 100 k-means centroids fit onto the atomistic data. These same 100 centroids are then also used to generate state assignments for the backmapped trajectory to which we compare. Complete validation of the MSM with associated clustering plots and implied time scale analysis for lag time selection is provided in the Supporting Information. Shown in Figure 5a are the recovered time scales for the test set atomistic and test set backmapped data. Our backmapped trajectory reproduces the atomistic time scales within error for all recoverable processes. A result of using the same state assignments when building the original atomistic and backmapped MSMs is that the elements of the recovered eigenvectors characterize eigenfluxes between the same states. We can therefore measure the cosine similarity between these eigenvectors to quantitatively validate that these time scales correspond to the same physical processes between the original atomistic and the backmapped trajectories. Indeed, shown in Figure S12a in the Supporting Information, we confirm via this cosine similarity measure that the MSM eigenvectors are effectively identically recovered for the backmapped trajectory of the in-distribution test set.

Shown in Figure 5b is a comparison of the implied time scales between reference atomistic data, backmapped trajectories generated by our model, and the original CGSchNet CG simulation. For both the CG backmapped data and the original CG simulation, we normalize the implied time scales such that the dominant (slowest) process matches the reference atomistic data. We perform such a normalization to correct for the fact that CG models typically sample configurational landscapes at an accelerated rate compared to atomistic force fields due to the absence of explicit solvent and elimination of atomistic degrees of freedom that can cause the coarse-grained degrees of freedom to be accelerated by different scaling factors.^18−21,89^ This normalization also serves as a visual convenience when comparing deviations of time scales between the accelerated CG dynamics and the atomistic data. An alternative method to this rescaling approach that provides the same information would simply measure time scale ratios instead. We report once again overall excellent agreement of the implied time scales, within error, between our backmapped and reference atomistic trajectories in Figure 5b, with the exception of the fourth, and subsequent, time scales that are faster than our lag time of 5 ps and therefore below the resolution limit of our MSM. We also validate again, in Figure S12b in the Supporting Information by comparing the MSM eigenvector similarity, that these first three time scales indeed correspond to the same physical processes between these two data sets. The backmapped CG simulated data ultimately reflect the kinetics produced by the coarse-grained model, evidenced by the nearly identical time scales of the CG simulation and backmapping in Figure 5b. Nevertheless, upon normalization of the time scales, our excellent agreement for these generalization results suggest that the ratio of time scales between processes is preserved upon backmapping with our model.

**Figure 6:**
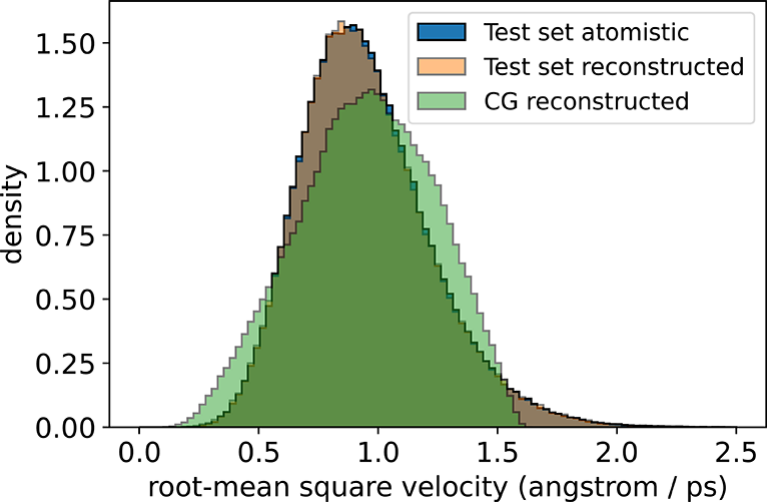
Comparison of atomistic and backmapped root-mean-square velocity distributions for ADP. Velocities are calculated atom-wise between sequential frames using a finite forward difference method and aggregated into a distribution over all atomic velocities. Velocities for the CG reconstructed data corresponding to the generalization set are rescaled by a constant factor (∼3.25) such that the mean of the CG reconstructed velocities matches the mean of the test set atomistic velocities.

As a result of conditioning our backmappings using previous atomistic configurations, we find our model is capable of successfully generating backmapped trajectories that reproduce intraframe root-mean-square velocity distributions (Figure 6). The distribution of velocities is an effective measure of the deviation of atomic coordinates between consecutive frames. Although our model is not explicitly trained to match intraframe velocities, the temporal coherence built into our network architecture and training procedure enables the trained network to accurately reproduce these velocity distributions. The excellent agreement we observe for the in-distribution data is enabled by the known and directly comparable spacing between frames. The temporal spacing between consecutive frames for the in-distribution test set is well-defined by the frame spacing of the atomistic reference simulation, i.e., 1 ps. However, the specific temporal spacing is more obscure for the generalization set, as a direct consequence of the CG dynamics. While most CG models target thermodynamic consistency with a yet higher resolution model or experimentally observed properties, kinetic consistency is typically neglected. In general, CG force fields effectively accelerate simulation dynamics as a consequence of smoothing the energy landscape and lowering energy barriers. However, the time scales for transitions between metastable states are typically not rescaled uniformly.^18−21,89^ To account for this inherent acceleration due to CG force fields, we rescale the velocity distribution of the backmapped generalization set data by a constant factor such that the mean of the velocity distribution matches the mean of the velocity distribution for the test set atomistic data. Applying this empirical correction, we see reasonable agreement in the shape of the velocity distributions between the backmapped generalization set data and the native atomistic data, suggesting for ADP here our backmapping model is capable of reconstructing realistic atomic velocities up to a constant scaling factor for data originating from CG force fields.

### 3.2 Chignolin

#### 3.2.1 Energetics

**Figure 7:**
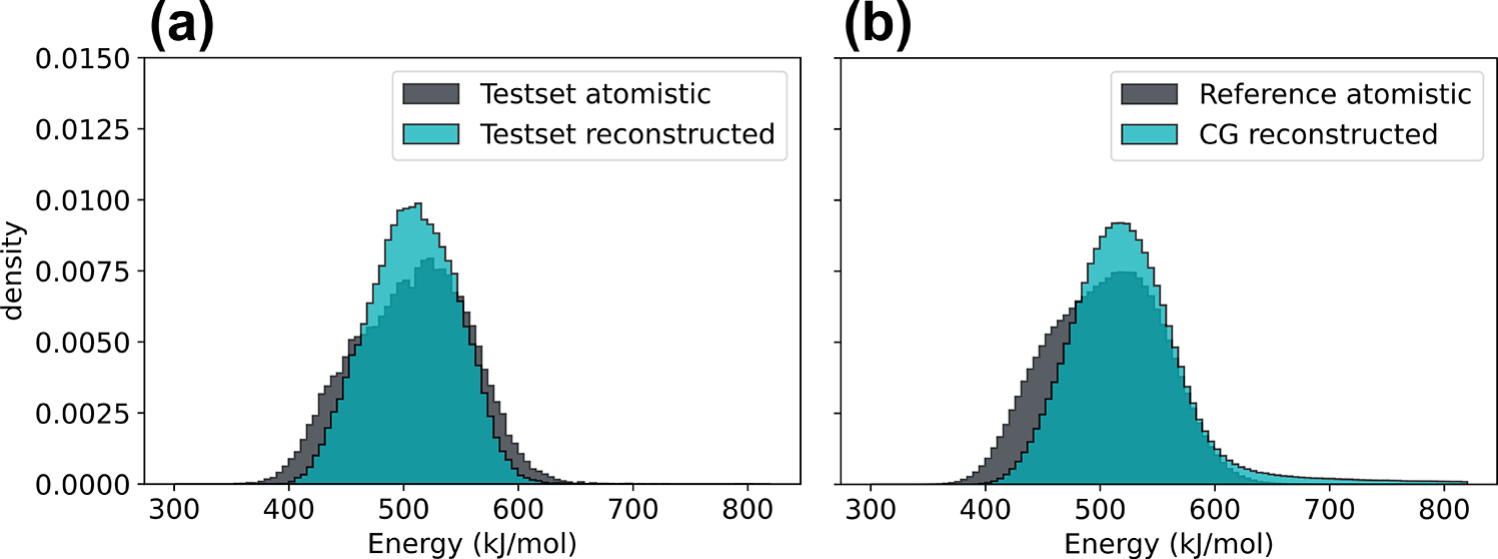
Potential energy distributions of atomistic and backmapped CLN trajectories for (a) the in-distribution test set and (b) the generalization set.

**Figure 8:**
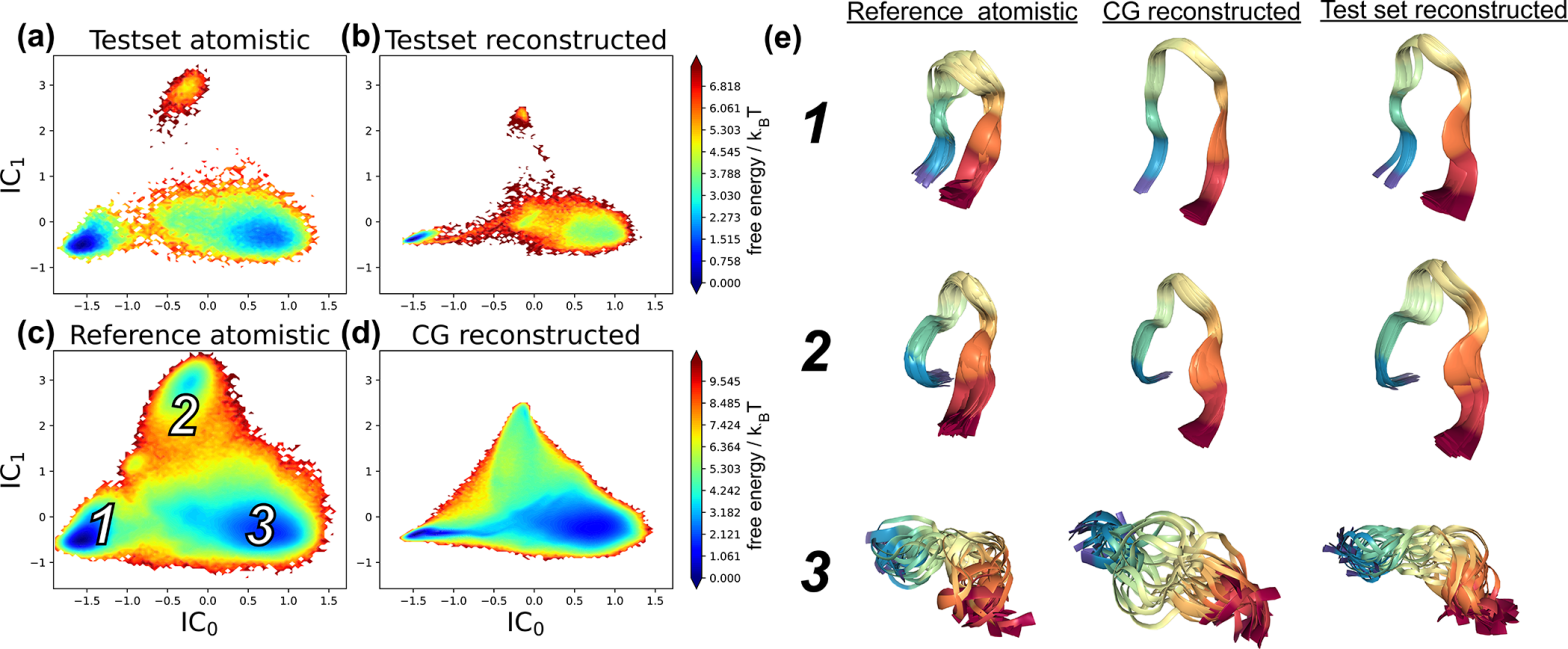
Comparison of the atomistic and backmapped MSM-reweighted Free Energy Surface (FES) for CLN. The FES for CLN is represented in the basis of the two leading nontrivial Independent Components (ICs) of a Time-lagged Independent Component Analysis (TICA) model fit to the reference atomic data set. Shown are the FES of the in-distribution test set atomistic (a) and backmapped (b) trajectories, alongside the reference atomistic data (c) and the corresponding backmapped CGSchNet simulation (d) from the generalization set. (e) Visualization for a collection of 25 superposed structures from the three metastable states taken from the reference atomistic data (c), the backmapped CG reconstructed data (d), and backmapped in-distribution test set trajectory (b).

The second molecular system we apply our backmapping method to is the miniprotin Chignolin (CLN), which is composed of 10 residues with 175 atoms from which we consider a coarse graining into the 10 α-carbons along the peptide backbone. Compared to ADP, the scale and complexity of CLN presents a more challenging test case for both our backmapping and accurate CG force field construction. We once again compare distributions of the internal energy as an indicator for structural similarity between reference atomistic and our backmapped data. Shown in Figure 7a is a comparison of the internal energies for the in-distribution test set atomistic and backmapped trajectories, while a comparison for the generalization set data is presented in Figure 7b. These results show excellent energetic overlap between the reference atomistic and our backmapped data for both the in-distribution and generalization sets. The internal energies recovered from the backmapped CGSchNet simulation in Figure 7b from the generalization test show longer high-energy tails compared to the in-distribution test in Figure 7a. The origins of these higher-energy reconstructed configurations can be explained by an examination of the bond lengths and angles revealing the presence of very slightly contracted bond length distributions in the generalization set compared to the in-distribution test set (Figures S23–S26 in the Supporting Information). Our backmapped trajectories nevertheless show overall excellent agreement in these local intermolecular features and energetics and therefore also produce visually convincing atomistic reconstructions (Figure 8e and Figure S6 in the Supporting Information).

#### 3.2.2 Thermodynamics

We construct Free Energy Surfaces (FES) using the basis recovered from Time-lagged Independent Component Analysis (TICA)^82,90−92^ to compare thermodynamic similarity between reference atomistic data and our backmapped trajectories. Unlike ADP, there are no simple intuitive variables capable of compactly representing the CLN FES, so we instead use TICA as a dimensionality reduction technique to extract a low-dimensional representation for the CLN phase space. Using our entire reference atomistic data set, we first learn a TICA embedding into the first two nontrivial Independent Components (ICs) using all 45 pairwise α-carbon distances as features.^75,76,93,94^ This learned TICA model provides us with a fixed basis set we use for constructing an FES in these two leading ICs for the atomistic and backmapped data from both our in-distribution and generalization data sets.

Shown in Figure 8 is a comparison of these CLN FESs for the in-distribution test set atomistic (Figure 8a) and backmapped (Figure 8b) trajectories, alongside the generalization set reference atomistic FES (Figure 8c) and the FES obtained from backmapping a CG simulation performed with CGSchNet^75^ (Figure 8d). In each case, the backmapped FES recovers the presence of the three primary metastable states in CLN and produces visually identical atomic reconstructions compared to the atomistic reference (Figure 8e). We notice our backmapped FES is contracted near regions corresponding to the folded state (labeled 1) and the misfolded state (labeled 2) compared to the unfolded ensemble (labeled 3). Correspondingly, backmapped structures extracted from the folded and misfolded states display slightly less configurational variability compared to atomistic reference than structures visualized from the unfolded ensemble (Figure 8e). We suspect this is due to a loss-conserving strategy by the network: comparatively less loss is sacrificed by predicting a (nearly) fixed configuration for CG structures corresponding to folded and misfolded states compared to the benefit of capturing the diversity of possible structures in the unfolded ensemble. This results in more comprehensive coverage of the unfolded ensemble in our backmapped data compared to the folded and misfolded states. We also notice that the misfolded state is noticeably less stable in the generalization set backmapping (Figure 8d) compared to the reference atomistic data (Figure 8c). Indeed, we find this property to be a reflection of the misfolded state being inherently less stable in the original CG simulation as well (Figure S27 in the Supporting Information). While our backmapping method does a reasonable job of reproducing the atomistic FES, we find that it is ultimately limited by the underlying accuracy of the CG simulation that we backmap.

#### 3.2.3 Kinetics

We evaluate kinetic similarity between reference atomistic data and our backmapped trajectories by comparing similarity of MSM-recovered implied time scales and processes. We perform state space discretization for our MSMs within the same two-dimensional TICA basis used to construct our FES. Data from the reference atomistic trajectories in this two-dimensional TICA space is fit to determine 150 k-means centroids identifying the state space decomposition. We then use these same 150 centroids to generate state assignments when building separate MSMs for other atomistic and backmapped data within the in-distribution and generalization data sets. Using the same TICA projection and set of cluster centers between MSMs ensures direct comparability of the recovered eigenvectors and allows us to determine the similarity of the recovered processes. We use the same methodology as with ADP here for CLN for quantifying the similarity of processes by measuring the cosine similarity of the MSM eigenvectors. In the cases where not all 150 states are utilized, we construct MSMs using only occupied states and compute cosine similarity using only this subset of mutually occupied states between the two MSMs being compared. Using this approach, we can quantitatively ensure the that implied time scales in fact correspond to the same physical processes between the two data sets. As with ADP, complete details on MSM construction and validation are provided in the Supporting Information.

**Figure 9:**
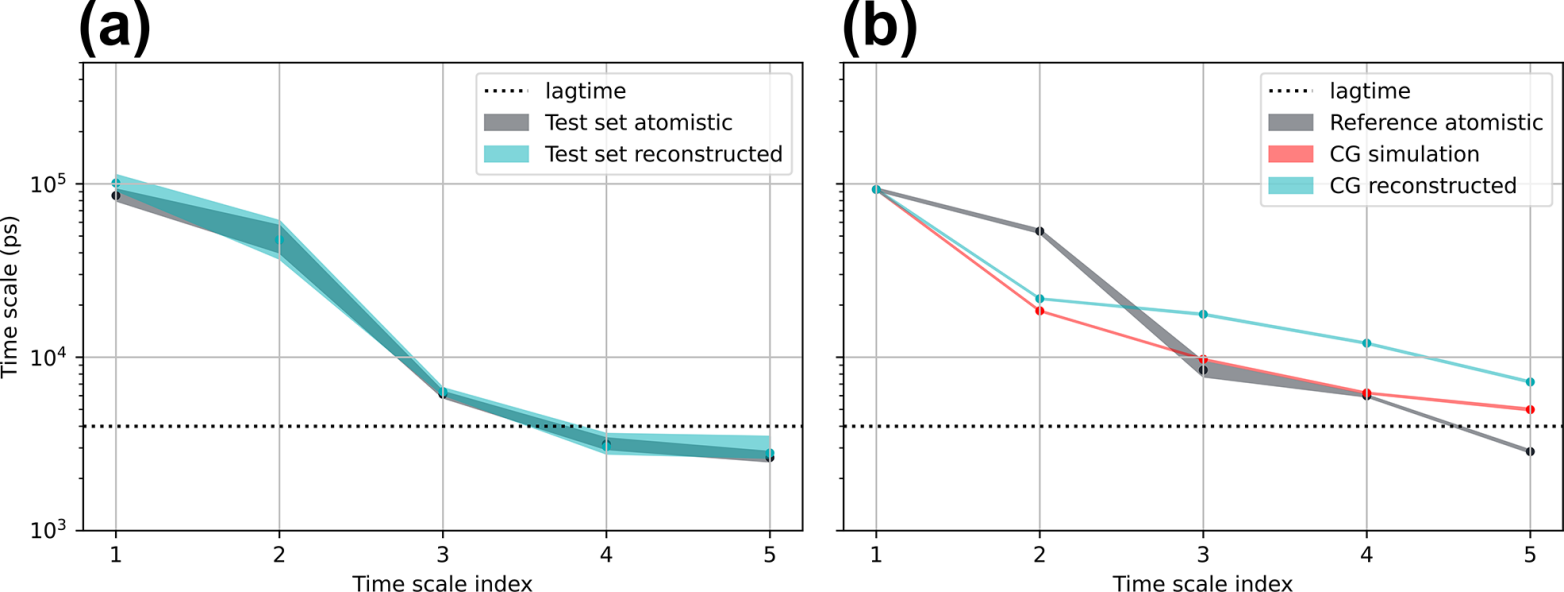
Implied time scales of atomistic and backmapped CG trajectories for CLN. (a) Comparison of implied time scales from atomistic and backmapped trajectories for the in-distribution test set. (b) Implied time scales for the reference atomistic data, the original CGSchNet simulation, and the backmapped CGSchNet simulation. Time scales for the original CG simulation and backmapped data are normalized such that the dominant (slowest) processes match the slowest reference atomistic time scale.

Presented in Figure 9a is a comparison of implied time scales between atomistic and backmapped trajectories for the in-distribution test set. For the in-distribution test set, our backmapped reconstruction precisely reproduces within error all implied time scales. The large gap between the second and third time scales suggests that the majority kinetic variance is captured in the first two processes, which we recover with >90% cosine similarity, and for the final remaining processes greater than our lag time—and therefore the only other resolvable process by our MSM—we also recover with ∼80% cosine similarity (Figure S28a in the Supporting Information). A comparison of implied time scales for the generalization set between the reference atomistic data, backmapped CGSchNet^75^ simulation, and the original CG simulation is shown in Figure 9b. We follow the same approach here as with ADP, where we normalize the implied time scales for data generated from CG simulations by a constant factor such that their slowest time scale matches the slowest time scale from the reference atomistic data. This normalization accounts for the inherently accelerated dynamics of CG simulations and enables us to effectively compare the ratio of implied time scales.^18−21,89^ The ratio between the first two time scales is poorly recovered for both the backmapped data and the original CG simulation compared to the reference atomistic data. Comparison of the cosine similarity between these processes reveals the first processes are recovered with ∼60% similarity for both the backmapped data and the CG simulation, while the second processes are recovered with also ∼66% similarity for the backmapped data and ∼90% similarity for the original CG simulation (Figure S28b in the Supporting Information). While the ratio in the time scales for the faster third and fourth processes seems to be better conserved in the original CG simulation, our backmapped data actually recover these processes with slightly better similarity than the original CG simulation (Figure S28b in the Supporting Information). The fact that our method precisely recovers kinetics for the in-distribution data but can only approximately recover kinetics when backmapping real CG simulated data confirms that our method produces backmapped trajectory data that are largely a reflection of the kinetics expressed in the underlying CG data.

**Figure 10:**
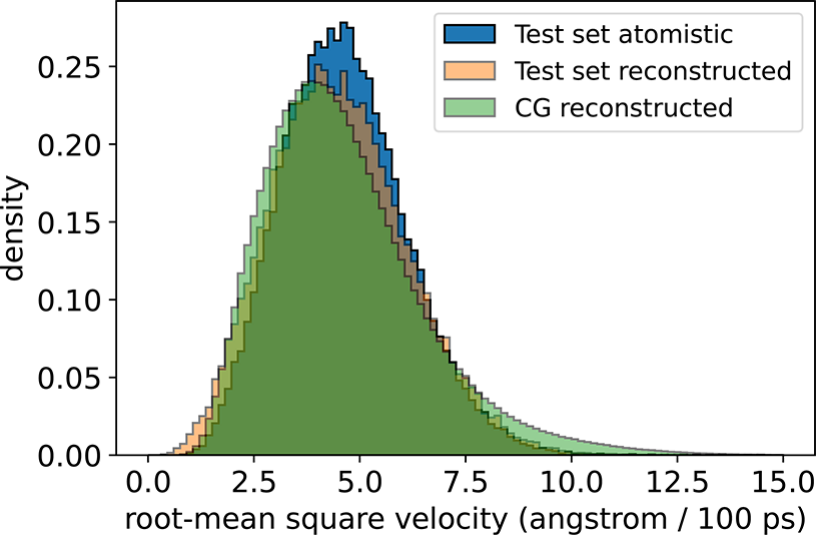
Comparison of atomistic and backmapped root-mean-square velocity distributions for CLN. Velocities are calculated frame-by-frame using a finite forward difference method and represented in angstroms/100 ps, as 100 ps is the native temporal spacing between frames in the reference atomistic data.^76^ The backmapped generalization set velocities are rescaled by a constant factor (∼17.81) such that the mean of the CG reconstructed velocities equals the mean of the test set atomistic velocities.

Last, we compare velocity distributions as an indicator of temporal coherence between sequential frames in our backmapped reconstructions. Shown in Figure 10 is a comparison of frame-by-frame root-mean-square velocity distributions for the reference and backmapped in-distribution test set data alongside the backmapped CGSchNet simulation data. For the in-distribution test set, the velocities are an excellent match between the reference and backmapped data. Mimicking our approach with ADP, to account for the inherently accelerated dynamics of the CGSchNet force field, we use a constant factor to rescale the atomic velocities such that the mean of the backmapped generalization set velocities matches the mean of the test set atomistic velocities. After applying this constant scaling, we notice excellent agreement in the shape of the velocity distributions between the backmapped generalization set data and the original atomistic data. We note that the scaling factor used to correct the CLN velocities (∼17.81) is much larger than the scaling factor used for the ADP velocities (∼3.25), suggesting a more substantial acceleration of backmapped CLN dynamics due to the CG force field compared to ADP. This relative speed-up of the backmapped CLN CG simulation could be attributed to the more severe coarse-graining of CLN from 175 atoms to 10 beads (17.5× reduction) compared to the ADP model from 22 atoms to six beads (∼3.67× reduction). This more dramatic reduction in the degrees of freedom upon coarse-graining for the CLN model could result in the CLN CG simulation operating in a comparatively “smoother” free energy surface than ADP and therefore leading to the relatively faster atomic motions identified by the velocity distributions.

## 4 Discussion and Conclusions

We present in this work a data-driven and temporally coherent scheme for backmapping CG trajectories into atomistic resolution. Our approach trains a conditional variational autoencoder (cVAE) to reconstruct atomistic detail given the target CG configuration and the previous atomistic structure. Our method is showcased here to backmap two biomolecular systems: alanine dipeptide (ADP) and the miniprotein chignolin (CLN)—systems that are frequently used as the test bed in the demonstration of new methods in molecular dynamics simulation.^43,75,95−97^ We train our model using a reference atomistic trajectory which we coarse-grain *post hoc* to produce exemplar pairs of atomistic and CG configurations (Figure 1a). We tested our backmapping method on both in-distribution data generated from backmapping a CG trajectory produced by coarse-graining held-out atomistic data (Figure 1b) and out-of-distribution data generated from a real CG simulation performed using CGSchNet.^75^ We evaluate the performance of our model in terms of capability to reproduce structural, thermodynamic, and kinetic properties of reference atomistic systems. To this end, structural similarity is probed by comparing distributions of potential energies and local structural features, such as bond lengths and angles. Thermodynamic similarity is tested by analyzing free energy surfaces that are constructed in terms of collective variables. Kinetic agreement is tested by comparing implied time scales of processes identified by MSMs, while temporal coherence between consecutive frames is analyzed in terms of intraframe velocity distributions. Our model yields backmapped trajectories for the in-distribution test set that are in good agreement with atomistic data. Moreover, our model generalizes well, producing convincing atomic reconstructions for out of distribution data obtained from CGSchNet simulations. While we notice slightly better generalizability to CGSchNet simulations of ADP compared to the more complex CLN molecule, we find our method generates backmapped trajectories that largely maintain thermodynamic and kinetic properties reflected in the original CG simulation while also reconstructing atomistic velocities up to a constant scaling factor.

As coarse-grained models are typically designed to accelerate conformational space sampling, this can lead to legitimate coarse-grained configurations which correspond to under-represented atomistic training structures. Data-driven backmapping should therefore strive to effectively extrapolate on the atomistic training data and generate reasonable backmapped atomistic reconstructions. Our method in general performs well when backmapping coarse-grained models that express good phase space overlap between coarse-grained and atomistric resolutions, but if this phase space deviation becomes too large due to poor coarse-grained models, we may expect our backmapping to help heal these errors to some extent through training. However, there is a limit to this remediation, and at some point it must fail. As a result, errors in our backmapped reconstructions on the out of distribution generalization data can be attributed to both the performance and expressivity of our backmapping cVAE model compounded with biases and inaccuracies of the CGSchNet force field used to generate the CG trajectories. In the case of ADP, the highly accurate CGSchNet model leads to overall good backmapping generalization performance. For CLN, the CGSchNet model is comparatively lower-fidelity, which we find results in slightly poorer generalization by our backmapping model in comparison to ADP.

Future work will strive to improve upon transferability to different coarse-grained mappings, data efficiency, and training/inference routines of our method. As a possible approach to avoid training separate bespoke models for different coarse-grained mappings of the same atomistic structure, a single set of atomistic simulation data could be used to train a model capable of backmapping a number of different coarse-grained representations. One idea in this direction is a hierarchical backmapping approach which involves an autoregressive component where separate prediction heads are tasked with reconstructing intermediate coarse-grained representations at progressively more detailed resolutions, conditioned on the preceedingly generated configurations until the final atomistic structure is produced. Transferability in this aspect would eliminate the need for retraining separate large models for each system through utilizing only a fixed set of atomistic simulation data for training. Currently, the backbone of our model primarily uses convolutional neutral networks (CNNs) operating on voxelized representations that are converted to and from Cartesian coordinates. Using explicitly covariant network architectures,^98−100^ such as those employed in the backmapping scheme by Wang et al.,^43^ can lead to superior data efficiencies without the need to train with random rotations and the potential to massively reduce the network size and memory requirements compared to voxelizations improving scalability. This approach specifically uses graph neural networks with *E*(3) equivariant operations to perform generative frame-by-frame backmapping. Temporal coherence could possibly be incorporated within this framework by including preceding trajectory configurations as conditioning variables for the structure generation processes. Our approach is also currently designed for configurational backmapping of superatoms into high resolution atomistic detail, but our scheme for achieving temporal coherence could also be extended to different definitions of coarse-graining such as the dynamical coarse-graining employed in molecular latent space simulators by Ferguson and co-workers.^101^ Training routines could also be augmented to incorporate more inductive biases that may benefit backmapping, such as (i) better emphasizing sparsely populated regions of configurational space, which could be accomplished by accompanying training samples with thermodynamic or dynamical path weights; (ii) an autoregressive training protocol that could be employed to improve the temporal coherence by using a recurrent approach to predict multiple consecutive frames for each forward pass; (iii) further encouraging the model to utilize knowledge of preceding trajectory frames by augmenting the training loss to incorporate information that is explicitly based on velocities or higher order time derivatives.

## Notes

A complete PyTorch implementation of our model with associated training routines, data, and pretrained model weights for ADP and CLN along with Jupyter Notebooks demonstrating the energetic, thermodynamic, and kinetic analyses performed in this work is provided via the Materials Data Facility (MDF)^102,103^ at DOI: 10.18126/tf0h-w0jz.^52^

## Supporting Information Available

Additional methodological details including neural network architecture and training specifications for our backmapping cVAE and CGSchNet models and additional figures accompanying ADP and CLN structural, thermodynamic, and kinetic analyses (PDF)
