# Supplementary material for: Temporally Coherent Backmapping of Molecular Trajectories From Coarse-Grained to Atomistic Resolution
Source: J Phys Chem A. 2022 Nov 23;126(48):9124–39. doi: 10.1021/acs.jpca.2c07716 (PMC9743211; doi:10.1021/acs.jpca.2c07716)
Supplement: Supplementary file 1 — jp2c07716_si_001.pdf [file jp2c07716_si_001.pdf]

SUPPORTING INFORMATION: Temporally Coherent Backmapping of Molecular Trajectories From Coarse-Grained to Atomistic Resolution

Kirill Shmilovich,[*,†] Marc Stieffenhofer,[‡] Nicholas E. Charron,[¶,‖] and Moritz Hoffmann[§]

†*Pritzker School of Molecular Engineering, University of Chicago, Chicago, Illinois 60637, United States*

‡ *Max Planck Institute for Polymer Research, Mainz 55128, Germany*

¶ *Weiss School of Natural Sciences, Department of Physics and Astronomy, Rice University, Houston, Texas 77005, United States*

§ *Fachbereich Mathematik und Informatik, Freie Universität Berlin, Berlin 14195, Germany*

‖ *Department of Physics, Freie Universität Berlin, Berlin 14195, Germany*

E-mail: kirills@uchicago.edu

1 Supporting Methods

1.1 cVAE architectural details and training settings

We discuss here details and hyperparameters in our cVAE architecture and training setup, with all code and datasets publicly available at DOI:10.18126/tf0h-w0jz.[1] Typical VAEs are composed of an encoder-decoder architecture where the encoder process an input into a fixed dimensional latent code that the decoder purposes to generate reconstructions of the input. Conditional VAEs (cVAEs) differ from this paradigm by defining an auxiliary conditional variable alongside the reconstruction target, which is passed to the decoder alongside the latent code when generating reconstructions. This cVAE framework enables the decoder alone to act as a generative model provided a conditional variable as input and a randomly sampled latent code as a source of generative noise.

In our application, we strive to produce temporally coherent backmappings by incorporating as input previous atomistic configurations AA^{t-1} alongside the current coarse grain CG^t configuration when generating reconstructions $\hat{\mathrm{AA}}^t$. This temporal coherence is achieved in our cVAE framework by defining the reconstruction target $\mathbf{y} = \mathrm{AA}^t$ and the conditional variable as the tuple of configurations $\mathbf{c} = (\mathrm{CG}^t, \mathrm{AA}^{t-1})$.

Our model operates on 3D spatially voxelized particle densities delineated for each particle which we can readily transform to/from Cartesian coordinates (cf. Sec. 2.1 of the main text). Presented in Fig. S1 is an illustration of our cVAE architecture and data flow exemplified for the alanine dipeptide (ADP) molecule. Beginning in the top left of Fig. S1, the input to our encoder $\mathbf{x}$ are the three configurations (AA^{t-1}, CG^t, AA^{t-1}) voxelized and concatenated along their particle dimension $\mathbf{x} = [\mathrm{VOX}^{\mathrm{AA}^{t-1}} || \mathrm{VOX}^{\mathrm{CG}^t} || \mathrm{VOX}^{\mathrm{AA}^{t-1}}] \in \mathbb{R}^{d \times d \times d \times (2N+n)}$, where d is the number of grid points used for the spatial discretization, N is the number of atoms within each atomistic configuration and n is the number of beads within each CG configuration. For this exemplar case of ADP shown here we use a spatial discretization of $d = 12$ with $N = 22$ and $n = 6$, yielding an input tensor with dimensionality

$\mathbf{x} \in \mathbb{R}^{12\times12\times12\times50}$. The different components of the input tensor are color-coded to designate the configurations associated with the conditional variable (CG^t, AA^{t-1}) (light blue) and the reconstruction target AA^t (orange).

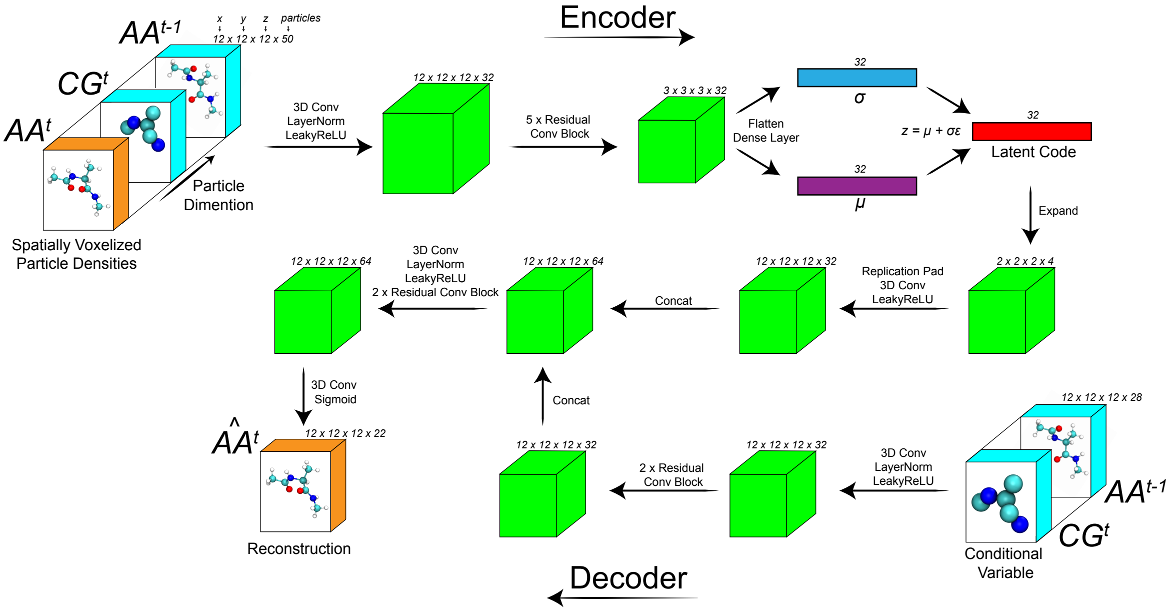

Figure S1: Schematic illustration of our cVAE architecture and data flow exemplified with particle dimensions above each tensor corresponding to our model trained on ADP.

The encoder processes the input $\mathbf{x}$ using a series of (residual) CNN modules. Each arrow in Fig. S1 represents a series of operations performed on a representation with the resultant tensor dimensionality shown above the subsequent block. For example, the first arrow in the top left of Fig. S1 indicates a series of computations on the input $\mathbf{x}$ involving a 3D convolution, followed by a LayerNorm and lastly a LeakyReLU activation yielding an intermediate representation with dimension $12 \times 12 \times 12 \times 32$. Here, $d_{hidden} = 32$ represents the hidden channel dimensionality of the network. The next step within the encoder involves sequential application of a $5\times$ series of residual convolutional blocks. In some cases we use different hidden channel dimensions for the encoder $d_{hidden}^{Encoder}$ and the decoder $d_{hidden}^{Decoder}$. A schematic illustration of our residual convolution block is shown in Fig. S2. In the case where the dimensionality of the input changes within the residual convolutional block, instead of a

simple identity operation the residual is accommodated to the appropriate dimensionality using max pooling and 1x1 3D convolutions. A variety of strides and kernel sizes are used within the five sequential residual convolutional blocks of the encoder to yield a representation with dimensionality $3 \times 3 \times 3 \times 32$. This representation is then flattened into a $3 \times 3 \times 3 \times 32 = 864$-dimension vector which is processed with two separate dense layers to yield two 32-dimensional representations corresponding to the mean $\boldsymbol{\mu}$ and standard deviation $\boldsymbol{\sigma}$ of the cVAE latent space. At training time, the latent code $\mathbf{z}$ is then sampled from $\boldsymbol{\mu}$ and $\boldsymbol{\sigma}$ using the reparamterization trick $\mathbf{z} = \boldsymbol{\mu} + \boldsymbol{\epsilon} \odot \boldsymbol{\sigma}$ where $\boldsymbol{\epsilon}$ is sampled from an isotropic Gaussian $\boldsymbol{\epsilon} \sim \mathcal{N}(\mathbf{0}, \mathbf{I})$. Here, the dimension of the latent space is $d_{latent} = 32$ such that $\mathbf{z} \in \mathbb{R}^{d_{latent}=32}$.

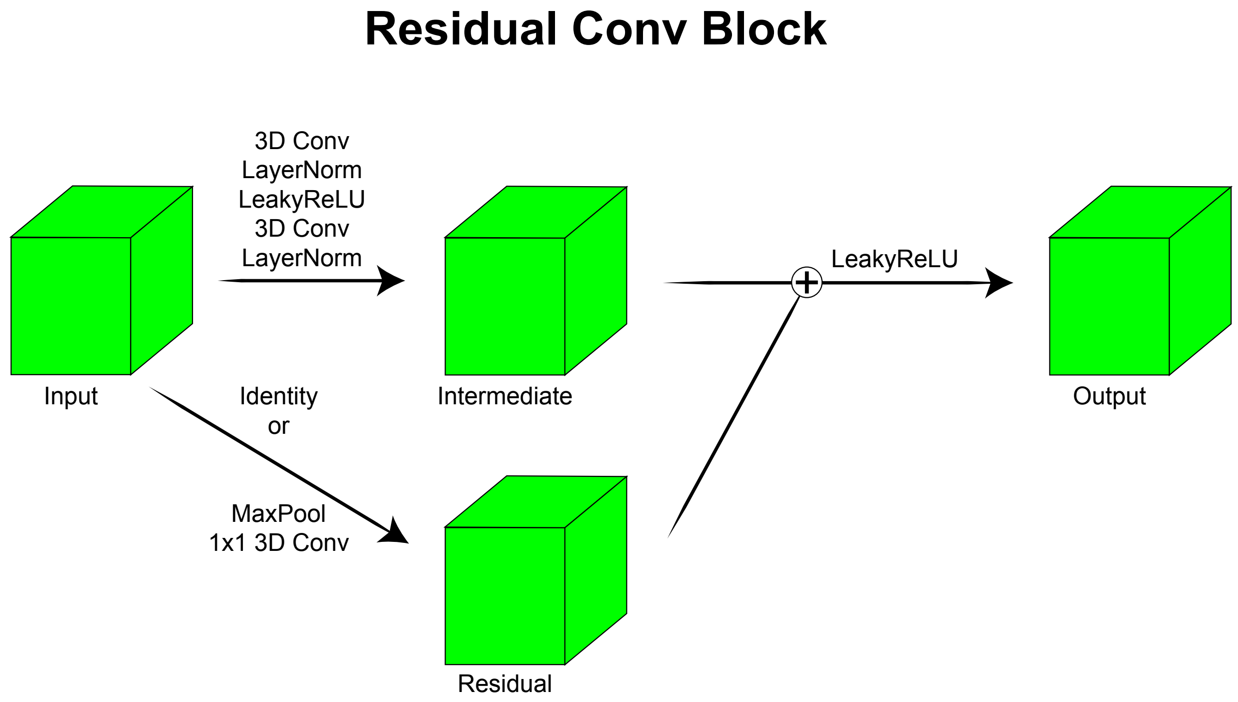

Figure S2: Schematic illustration of residual convolutional block used within our cVAE architecture.

Following extraction of the latent code $\mathbf{z}$ from the encoder, the decoder is passed the latent code $\mathbf{z}$ together with the conditional variable $\mathbf{c} = [\mathrm{VOX}^{\mathrm{CG}^t} || \mathrm{VOX}^{\mathrm{AA}^t}]$ to generate the voxelized reconstruction $\mathrm{VOX}^{\hat{\mathrm{AA}}^t}$. We can ultimately transform this output voxelized representation $\mathrm{VOX}^{\hat{\mathrm{AA}}^t}$ into Cartesian coordinates $\hat{\mathrm{AA}}^t$ for downstream application using

the methods outlined in Sec. 2.1 of the main text. The decoder begins with separately processing $\mathbf{z}$ and $\mathbf{c}$, which are later concatenated and further processed to yield the target reconstruction. First, displayed in the bottom right of Fig. S1 the conditional variable $\mathbf{c}$ of dimension $12 \times 12 \times 12 \times 28$ (there are 28=22+6 particle channels for $\mathbf{c}$ due to the 22 atoms within the atomistic structure the 6 beads of the coarse grain structure) is processed using (residual) 3D convolutions into an intermediate $12 \times 12 \times 12 \times 32$-dimensional representation. Concurrently, the $d_{latent} = 32$-dimensional latent code $\mathbf{z}$ is reshaped into a 4D tensor of shape $2 \times 2 \times 2 \times 4$. This representation is then expanded using the PyTorch replication padding and processed with 3D convolutions to generate a compatible $12 \times 12 \times 12 \times 32$-dimensional intermediate representation of the latent code. The processed conditional variable and latent code are then concatenated along the final dimensional to yield a $12 \times 12 \times 12 \times 64$-dimensional representation, which we further process with (residual) convolutions to yield the $12 \times 12 \times 12 \times 22$-dimensional output. The final particle-dimension of this output tensor $\text{VOX}^{\hat{\text{AA}}^t}$ corresponds to the number of particles in the target atomistic configuration. A terminal sigmoid activation scales the voxel values between 0-1 and we interpret each channel as a voxelized particle density, which we can ultimately then collapse back into Cartesian coordinates $\hat{\text{AA}}^t$ as the complete atomistic reconstruction.

The training loss for our model described in detail in Sec. 2.3 of the main text is

$$\mathcal{L} = \mathcal{L}_{\text{VOX}} + \mathcal{L}_{\text{AA}} + \mathcal{L}_{\text{CG}} + \mathcal{L}_{\text{EDM}} + \lambda\mathcal{L}_{\text{ENERGY}} + \beta\mathcal{L}_{\text{KLD}}. \tag{1}$$

Initially during training we find that $\mathcal{L}_{\text{ENERGY}}$ can become dominatingly large, and possibly NaN due to numerical overflow, motivating us to incorporate the prefactor λ which we set to $\lambda = 0$ for an initial $t_{\lambda=0}$ training steps. After which point once the model learns to roughly, but consistently, localize atomic coordinates we slowly anneal λ from a value of $\lambda_{initial}$ to

λ_{final} over the course of n_λ training steps using the following exponential annealing schedule

$$\lambda^{(t)} = \begin{cases} 0 & t < t_{\lambda=0} \\ \lambda_{initial}(\frac{\lambda_{final}}{\lambda_{initial}})^{\frac{(t-t_{\lambda=0})}{n_\lambda}} & t_{\lambda=0} + n_\lambda > t \geq t_{\lambda=0} \\ \lambda_{final} & t \geq t_{\lambda=0} + n_\lambda \end{cases} \tag{2}$$

where $\lambda^{(t)}$ is the value of λ at training step t (not to be confused with $\mathrm{AA}^t/\mathrm{CG}^t$ which are the atomistic/CG configurations at frame index t in our dataset). To further help stabilize training once $\lambda > 0$ and the $\mathcal{L}_{\mathrm{ENERGY}}$ takes effect, we use gradient clipping to clip gradient norms to a maximum value of w_{clip}, and for the CLN model we also clamp the maximum possible value of the reconstructed energy $U^{\hat{\mathrm{AA}}^t}$ to be at most U_{max} effectively limiting the maximum achievable value of $\mathcal{L}_{\mathrm{ENERGY}} = \mathcal{L}_{\mathrm{ENERGY}}(U^{\mathrm{AA}^t}, \max(U^{\hat{\mathrm{AA}}^t}, U_{max}))$. The internal potential energy is calculated entirely differentiably with OpenMM[2] using the AMBER99SB-ILDN[3] force field for ADP and the CHARMM[4,5] force field generated with the CHARMM-GUI[6] for CLN.

The β prefactor to $\mathcal{L}_{\mathrm{KLD}}$ controls the weight of the KLD loss relative to the other reconstruction loss terms. To mitigate KL vanishing for the ADP model we employ a cyclic annealing schedule on β with a sigmoid increasing function as described in Ref.[7] We define the schedule for determining the value $\beta^{(t)}$ at each training step t as,

$$\beta^{(t)} = \begin{cases} [1 + \exp(-k(\mathrm{mod}(t, R) - \tau))]^{-1} & t < MR \\ 1 & t \geq MR \end{cases} \tag{3}$$

In Eqn. 3 M represents the number of cycles, R is the length in training steps of each cycle, and k and τ are parameters for width and center of the sigmoid, respectively. For our CLN model we simply maintain $\beta = 1$ throughout training as we find KL vanishing is not problematic. A possible explanation for why our model benefits from cyclic annealing for the ADP data set could be related to ADP trajectory frames being spaced by 1 ps com-

pared to the 100 ps trajectory frame spacing for the CLN data. As the previous atomistic configuration AA^{t-1} is provided to the decoder alongside the current CG configuration CG^{t} as the condition, a possible failure mode for the model would be for the decoder to simply reproduce a replica the previous atomistic frame $\hat{\mathrm{AA}}^{t} \approx \mathrm{AA}^{t-1}$ as a low-loss reconstruction when sequential atomistic configurations are nearly identical. In this situation the decoder is no longer generative failing to effectively purpose the latent $\mathbf{z}$ and resulting in KL vanishing $\mathcal{L}_{\mathrm{KLD}} \to 0$. For the CLN model we find KL vanishing is not a problem when simply setting β=1 throughout training, possibly due to atomistic configurations in sequential frames being sufficiently different from one-another as the trajectory frames for the CLN data are separated by 100 ps, instead of the 1 ps spacing in the ADP data.

Our ADP model contains ∼1.2M parameters and is trained on a singe NVIDIA RTX 2080Ti GPU, and our CLN model contains ∼4.9M parameters and is trained on two NVIDIA RTX 2080Ti GPUs using distributed data parallel as implemented in PyTorch Lightning.[8] We train our model until the $\mathcal{L}_{\mathrm{KLD}}$ loss term has approximately converged, suggesting the posterior latent space has equilibrated. Provided in Table S1 is a complete list of model and training setting hyperparameter values with corresponding descriptions for both our ADP and CLN models.

1.2 Coarse Grain CGSchNet Force Fields

Coarse grain force field models of ADP are assessed using a 5-fold cross-validation strategy. Model creation and training was done using force matching via TorchMDNet[9] and PyTorch. Each of the model hyperparameters are described in S2:

The following prior energy terms were used to construct a baseline force field and to constrain basic molecular structure for each model. These prior terms are summarized in S2

Table S1: ADP and CLN model and training setting hyperparameters with associated descriptions.

Hyperparameter	ADP	CLN
Mini-batch size	32	32
Learning rate	1.398×10^{-4}	4.233×10^{-4}
Voxelized grid discretization (d)	12	12
Width of Cartesian grid (r_{grid})	1.8	5.5
Gaussian width of voxelized particle density (σ)	0.05398	0.3076
Dimension of cVAE latent space (d_{latent})	32	64
$\mathcal{D}_{KL}$ weight schedule (β)	Cyclic annealing (Eqn. 3) with $k = 0.0025$, $\tau_0 = 10000$, $M = 4$ and $R = 25000$	None; β=1
Maximum potential energy value (in kJ/mol) during training (U_{max})	None	1×10^5
Number of initial training steps with prefactor $\lambda = 0$ to $\mathcal{L}_{\text{ENERGY}}$ ($t_{\lambda=0}$)	2×10^5	7.5×10^5
Initial value of λ when starting to anneal λ ($\lambda_{initial}$)	1×10^{-6}	1×10^{-6}
Final value of λ after anneal λ (λ_{final})	1.0	1.0
Number of steps to anneal λ (n_λ)	1.25×10^6	1.25×10^6
Gradient clipping value used after $t_{\lambda=0}$ training steps (w_{clip})	0.1	0.1
Encoder hidden channel dimension ($d_{hidden}^{Encoder}$)	32	32
Decoder hidden channel dimension ($d_{hidden}^{Decoder}$)	32	64
Number of training steps	~14.8M	~9.4M

Table S2: CGSchNet Model Hyperparameters

Hyperparameter	ADP	CLN
Interaction Blocks	2	2
Hidden Channels	128	128
Filters	128	128
Neighbor Cutoff	5 Angstroms	30 Angstroms
Filter Network Cutoff Function	None	Cosine Cutoff
Radial Basis Functions	20	300
Radial Basis Function Span	0 to 5 Angstroms	0 to 30 Angstroms
Radial Basis Function Type	Exponential Normal	Gaussian
Terminal Network Layers	1	1
Terminal Network Width	128	128
Activation Function	Tanh	Tanh
Embedding Strategy	Atomic Number	Amino Acid Identity[1]
Priors	Bonds, Angles	Bonds, Angles, Repulsions

and S3.

Table S3: CGSchNet Model Hyperparameters

Prior Type	Energy
Bonds	$\frac{k}{2}(r - r_0)^2$
Angles	$\frac{k}{2}(\theta - \theta_0)^2$
Repulsions	$\left(\frac{\sigma}{r_{ij}}\right)^6$

Following previous work,[10,11] the spring constants (k) for angles and bonds were fit directly from reference data distributions. For ADP, all physical bonds and sequential triplet angles were used. For CLN, all sequential CA bonds and triplet angles were used. For the CLN repulsions, all beads pairs outside of bonds were assigned to the repulsion set, and the excluded volumes (σ) were uniformly set to 3.5 angstroms for all pairs in the repulsion set.

Following the procedure in Chen et al,[12] each model was training on the *delta forces*; that is, each model was trained on the difference between the reference forces and the forces predicted by the prior model:

$$F_{Delta} = F_{Ref} - F_{Prior} \tag{4}$$

Separate training routines were adopted for each molecule, following the procedure described in Husic et al.[11] They are reproduced in S4 for convenience.

To assess the performance of each CGSchNet Model, implicit solvent Langevin simulations were carried out with the parameters described in S5.

For both ADP and CLN, the following BAOA(F)B Langevin scheme was used:

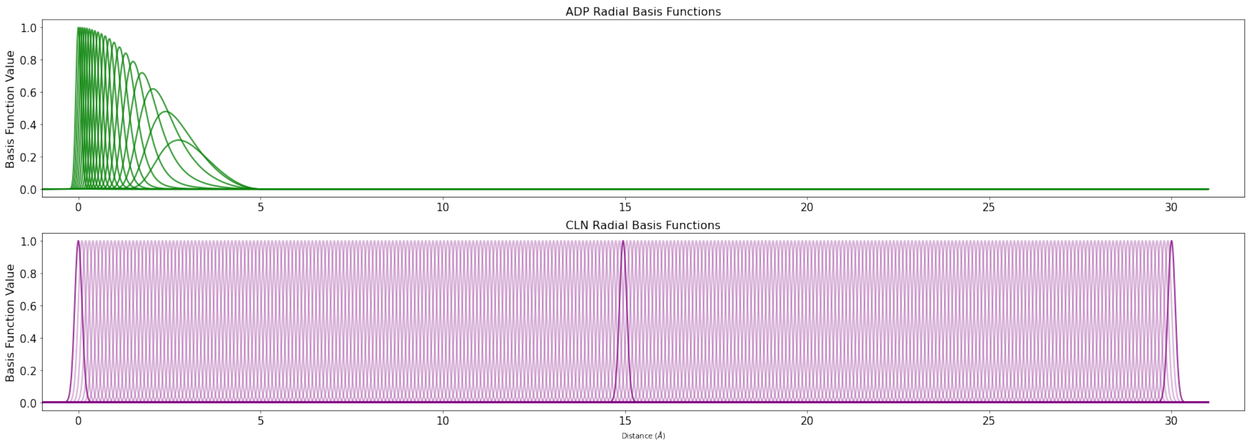

Figure S3: CGSchNet Radial basis function sets for ADP and CLN. For visual clarity, the first, middle, and last basis functions for CLN have been highlighted.

Table S4: Model Training Hyperparameters

Hyperparameter	ADP	CLN
Number of Epochs	100	100
Batch Size	512	512
Optimizer	ADAM	ADAM
Weight Initialization	Xavier Uniform	Xavier Uniform
Bias Initialization	Uniform($-\sqrt{k}$,$\sqrt{k}$)	0
Initial LR	0.0006	0.0001
LR Scheduler	ReduceLROnPlateau	None
Loss Function	MSE Force Matching	MSE Force Matching
Model Selection	Last Epoch	Minimum Average Validation Loss

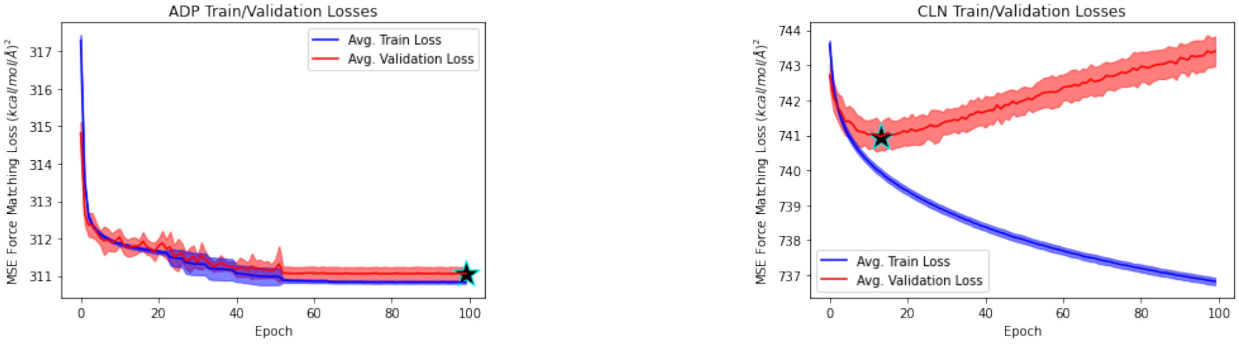

Figure S4: Epochal training and validation loss curves for the ADP and CLN CGSchNet models over 5 cross validation folds. The solid curves represent the average train/validation loss, while the shaded regions represent a moving standard deviation across the 5 models. The black star represents the epoch at which the final model was chosen.

Table S5: CG Simulation Parameters

Parameter	ADP	CLN
Integration Time step	4 fs	4 fs
Friction	1 ps^{-1}	1 ps^{-1}
Initial Structures	Random Sample From Train Set	Random Sample From Train Set
Number of Trajectories	100	1000
Temperature	300K	350K

1. $\bar{F} = -\nabla U(x_t)$
2. $v_{t+1} = v_t + dt\frac{\bar{F}}{m}$
3. $x_{t+1/2} = x_t + v_t\frac{dt}{2}$
4. $v_{t+1} = v_{t+1/2}\eta_v + dW_t\eta_n$
5. $x_{t+1} = x_{t+1/2}v\frac{dt}{2}$

where $\bar{F}$ is an average force predicted by averaging the force predictions from each model produced from cross validation,

$$\bar{F} = \sum_{i=1}^{\text{num folds}} f_i \tag{5}$$

η_v and η_n are velocity and noise scales respectively, and dW is a stochastic Wiener process. Forward and backward transitions through all major metastable states are observed in the CG simulations for both molecular systems.

1.3 Latent space embedding and samples

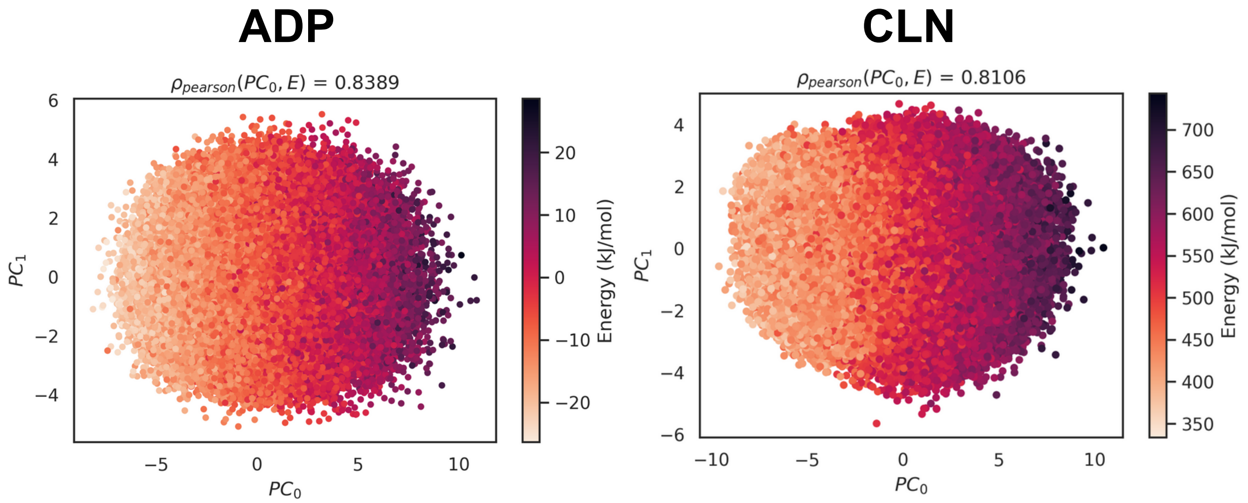

Figure S5: Projection of the full dimensional latent space embedding of all our **(a)** ADP and **(b)** CLN training data into the two leading principal components color-coded by the target atomistic configuration internal potential energy.

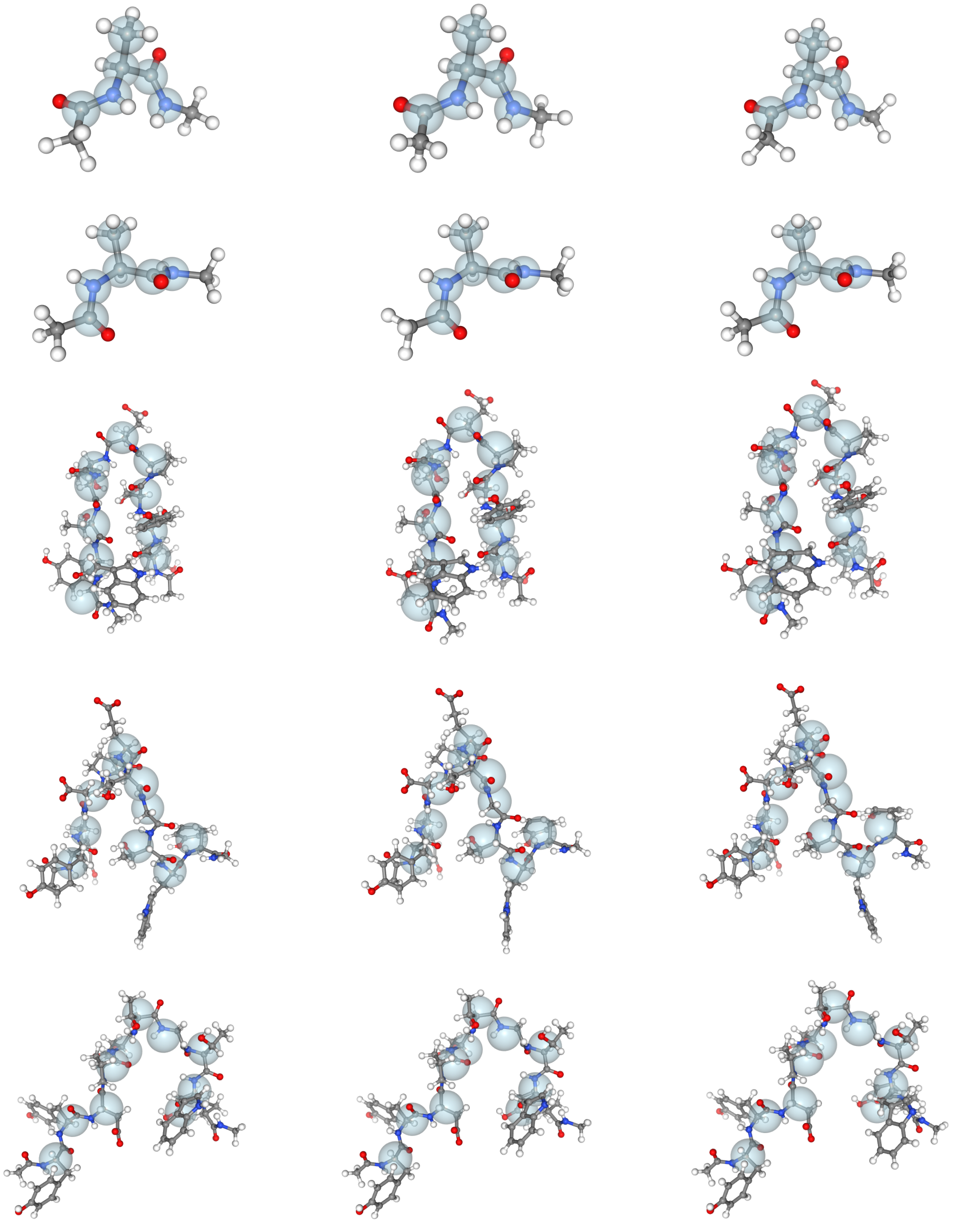

Figure S6: Atomic reconstructions generated from a fixed condition with different latent space instantiations. Each row represents a set of three atomic reconstructions for a fixed CG configuration. Faded blue spheres represent the input CG configuration overlaid on the atomic reconstruction. For ADP along the first three rows, the primary variation in these latent space samples are rotations of the methyl group hydrogens, as the rest of the peptide backbone and side chains are defined by the input CG configuration alone. For CLN along the final three rows, we notice the majority of configurational variation stems from side-chain motions along the C- and N-terminus.

1.4 Alanine dipeptide

Energetics

We notice overall excellent agreement between our atomistic and backmapped ADP trajectories for both the in distribution and generation test sets. This agreement results in nearly identical distributions of the internal potential energy (Fig. 3 of the main text). The backmapped data tends to slightly deviate by producing small high-energy tails. These rare high-energy configurations produced by our model are a result of a select few bond length and angle distributions that are moderately deformed compared to the reference atomistic data. We notice more pronounced deviations in the distribution of carbon-carbon bond lengths for the test set reconstructions (Fig. S7) compared to the generalization set reconstructions (Fig. S9), which results in a comparatively larger shift in the total energy distribution for the test set (Fig. 3 in the main text). Other notable discrepancies are atomic angles and bonds that include hydrogen atoms, and particularly terminal methyl group hydrogens. While the MD engine restrains all bond lengths involving hydrogen, our model can struggle to exactly reproduce these bond lengths due to the achievable resolution of the voxelized grid. The terminal methyl group of ADP is also entirely unspecified by the CG configuration, and methyl group hydrogen atoms are a significant source of variation rotating about the central carbon atom with relaxation times of $\sim$0.1-1 ps which is faster than the 1 ps spacing between consecutive frames.[13]

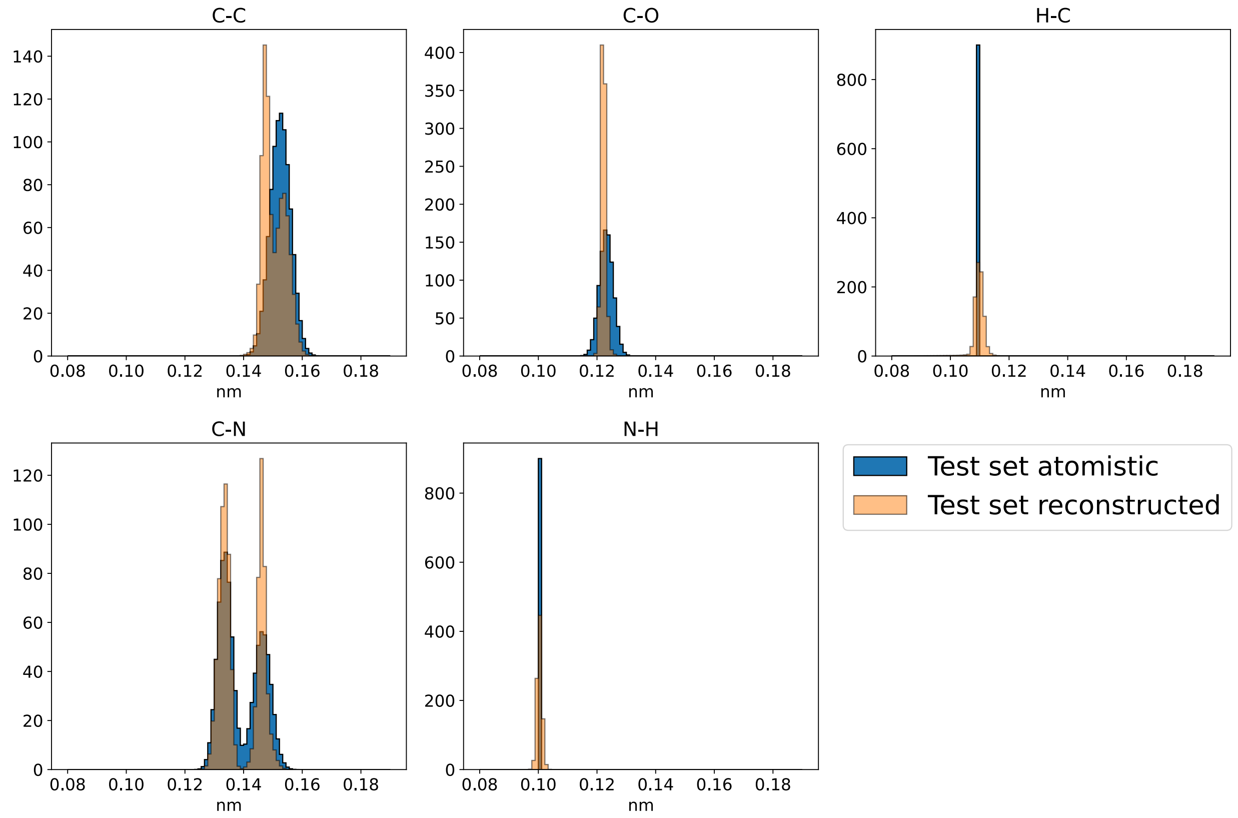

Figure S7: Bond length distributions delineated by element participation for the in distribution test set atomistic and backmapped trajectories for ADP.

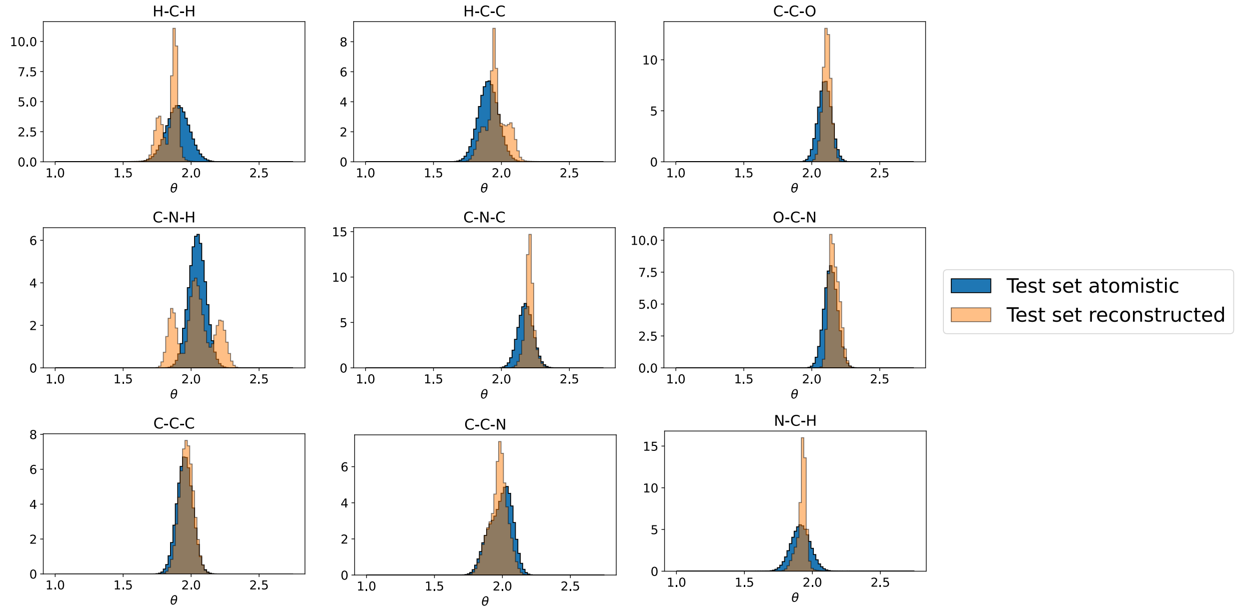

Figure S8: Atomic angle distributions delineated by element participation for the in distribution test set atomistic and backmapped trajectories for ADP.

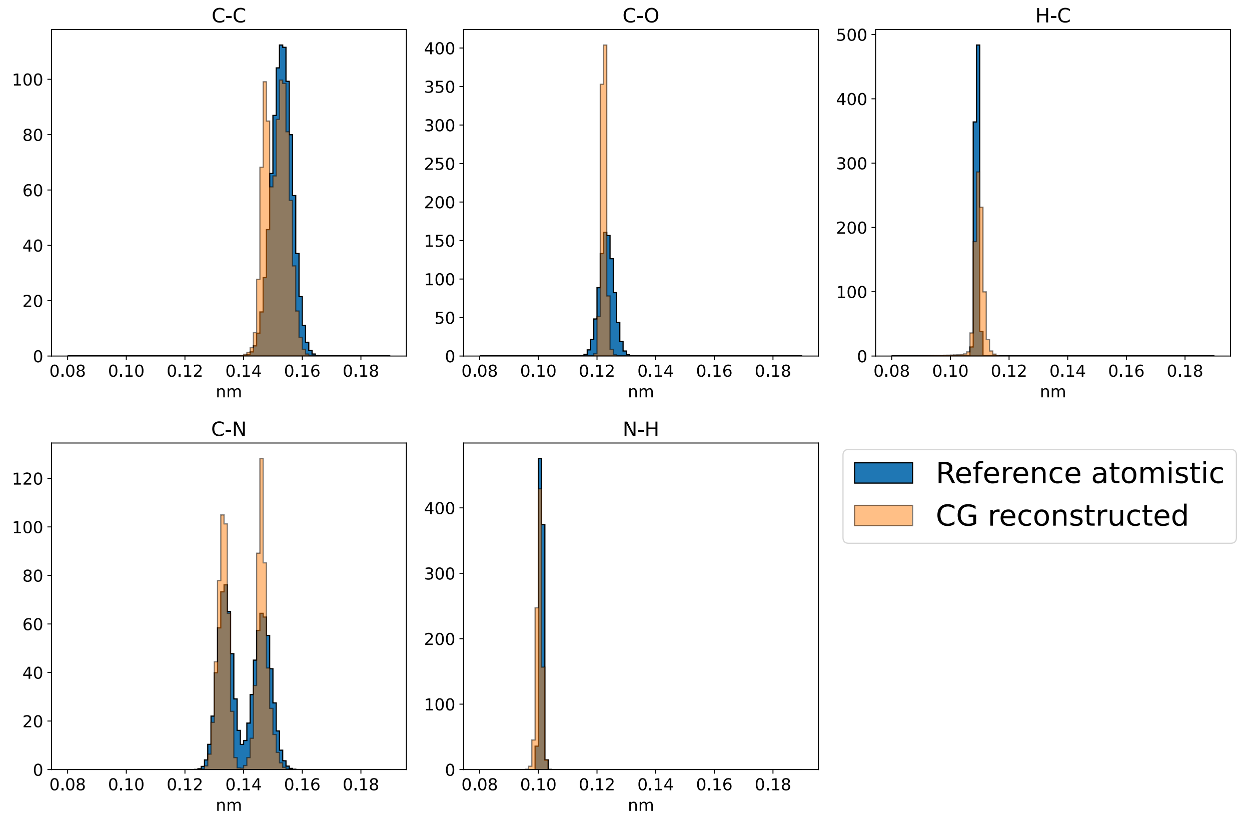

Figure S9: Bond length distributions delineated by element participation for the generalization test reference atomistic and backmapped CGSchNet trajectories for ADP.

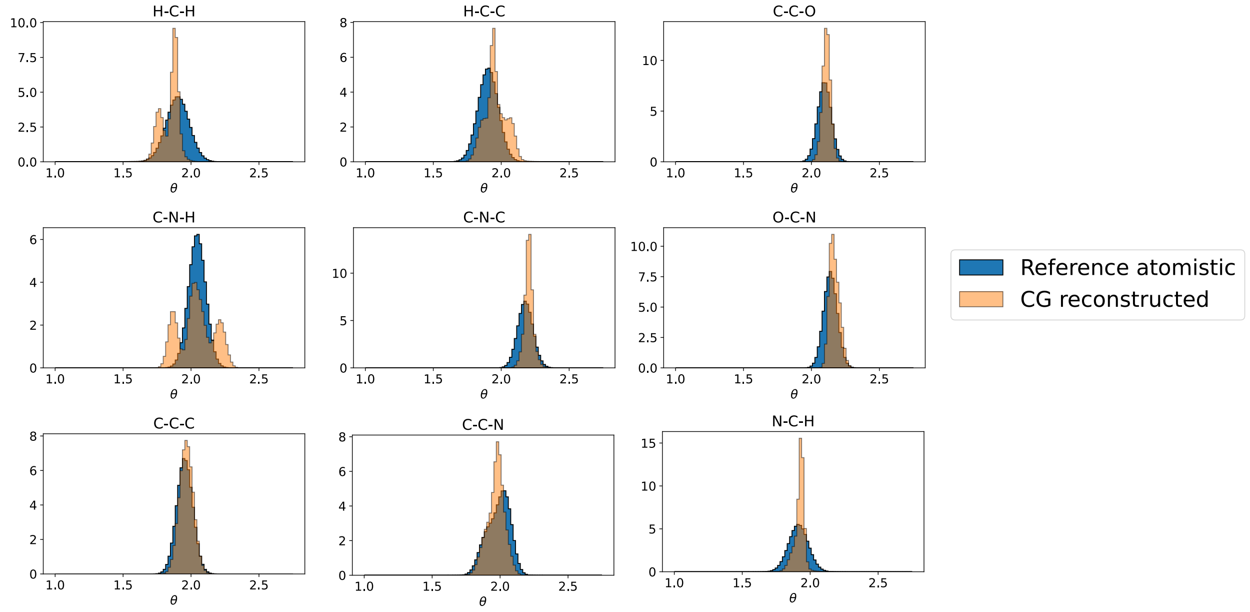

Figure S10: Atomic angle distributions delineated by element participation for the generalization test reference atomistic and backmapped CGSchNet trajectories for ADP.

Thermodynamics

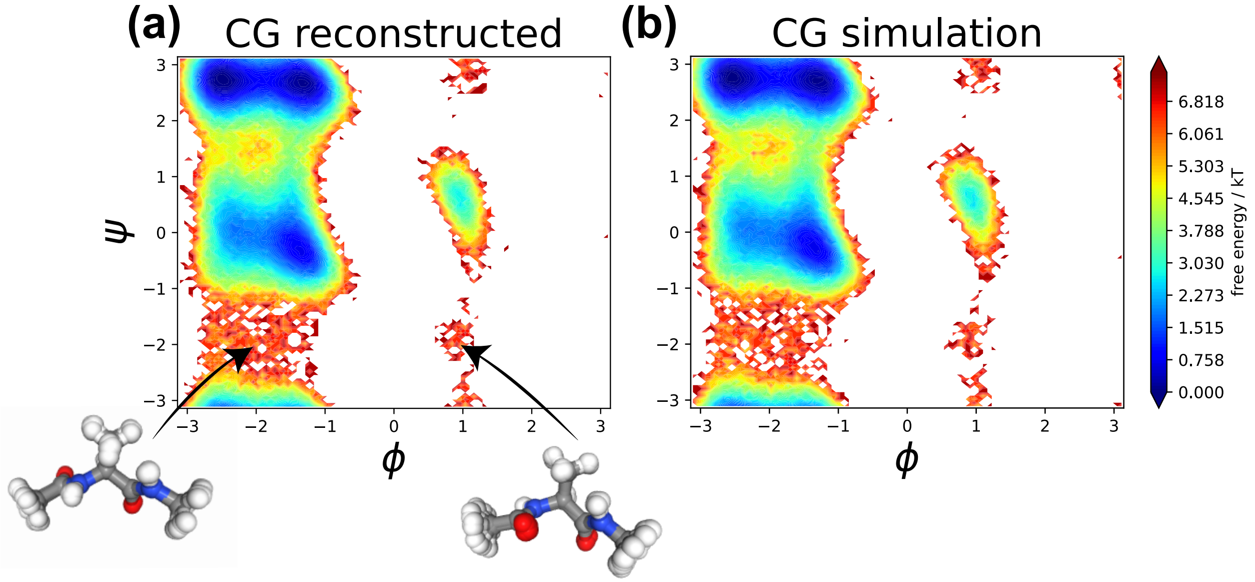

Figure S11: Comparison of MSM-reweighed ADP FES between the **(a)** the backmapped CG reconstructed trajectory and **(b)** the original CG simulation performed with CGSchNet. Insets show a superposition of seven configurations within the transition paths between select meta-stable states. Although these configurations are rarely seen in the atomistic training data, our model effectively generalizes to reliably reconstruct these high-energy configurations from the data simulated with CG force fields.

Kinetics

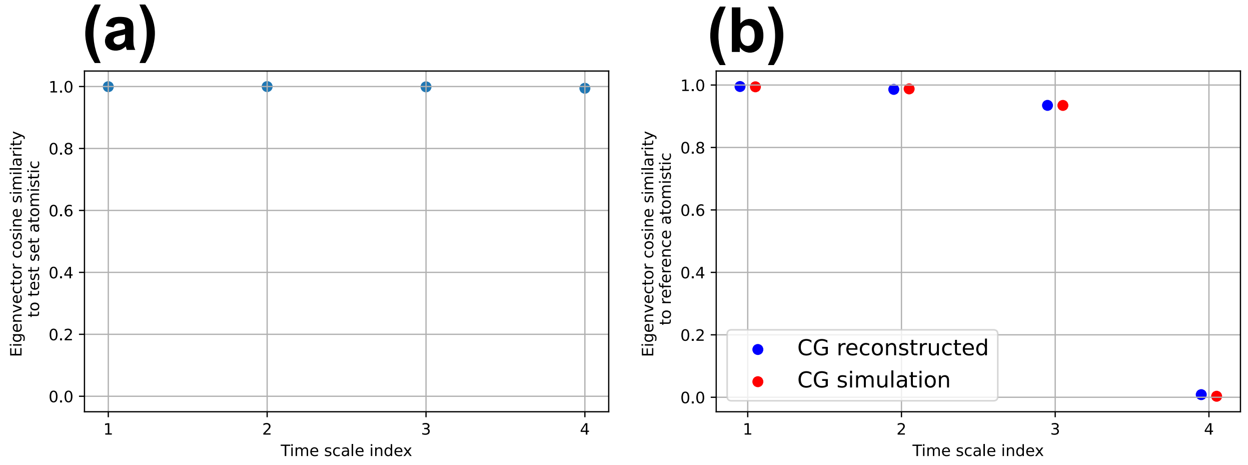

Figure S12: Cosine similarity between recovered MSM eigenvectors of atomistic and backmapped ADP trajectories. **(a)** The in distribution test set cosine similarity between atomistic and backmapped trajectories. **(b)** Cosine similarity between MSM eigenvectors of the backmapped data and the original CG data with respect to the reference atomistic data.

ADP MSM construction and validation

We construct Markov State Models (MSMs) to facilitate the comparison of the kinetics between the reference atomistic data and our backmapped trajectories in Sec. 3.1.3.

We construct MSMs for ADP over the phase space of the backbone ϕ,ψ angles as they are known to be good CVs for this system. When comparing kinetics between backmapped/CG data and atomistic data we perform k-means clustering for state space decomposition first on the atomistic dataset. We use these same k-means clusters to then produce state assignments for the backmapped/original CG data. Independent MSMs are subsequently fit to the atomistic and backmapped/original CG data from these cluster assignments to ensure direct comparability bewteen the recovered timescales and processes. Associated state space clustering plots, implied timescale analysis for lag time selection and Chapman-Kolmogorow (CK) tests for Markovianity for MSMs built on the different ADP datasets in this work are shown below.

For the in distribution test set we first perform k-means clustering using 100 centroids on the reference atomistic data, the same 100 clusters of which we then also use to build the backmapped in distribution test set MSM.

ADP in distribution test set atomistic

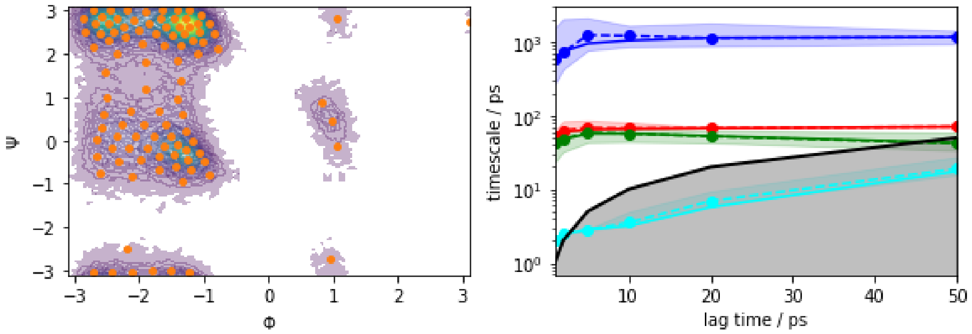

Figure S13: (left) State space clustering using 100 k-means centers fit to the in distribution test set atomistic data shown as orange dots over a contour of the data density. (right) Implied timescale analysis for MSM construction. Solid lines indicate the maximum likelihood MSM estimate, while the dashed lines show the mean implied timescale bounded by a 95% confidence interval. A lag time of 5 ps was selected.

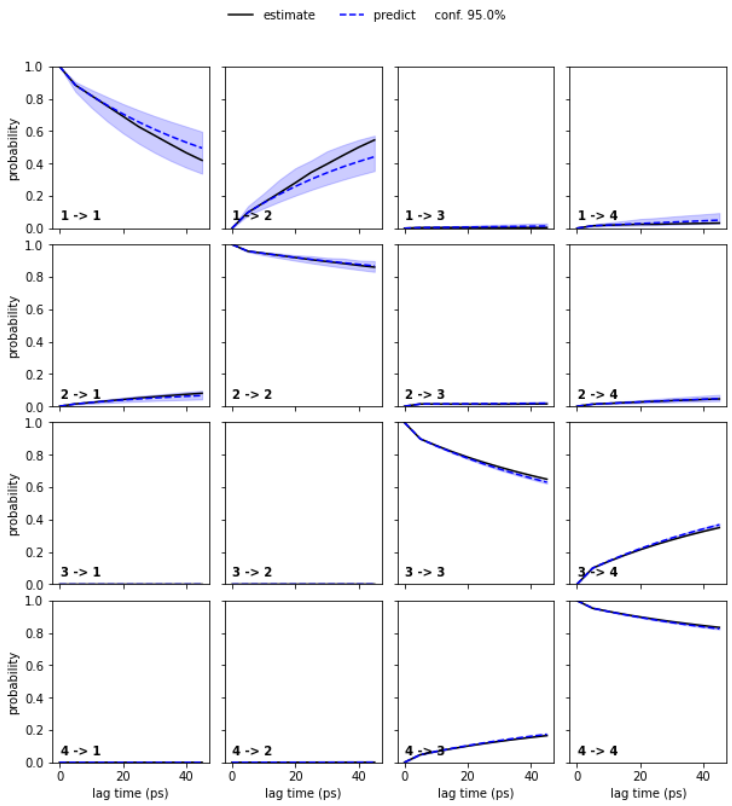

Figure S14: (Chapman-Kolmogorov (CK) test using 4 macrostates. Black lines indicate estimates, while the dashed blue lines are the predictions bounded by a 95% confidence interval)

ADP in distribution test set reconstructed

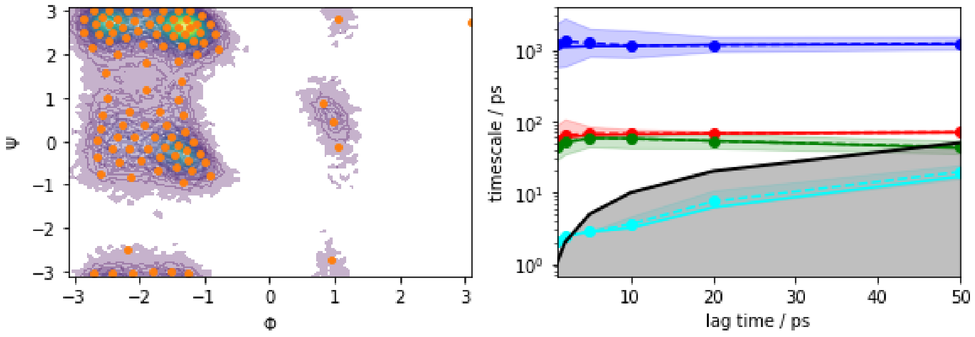

Figure S15: (left) State space clustering of the backmapped in distribution test set using 100 k-means centers taken from the in distribution test set atomistic data shown as orange dots over a contour of the data density. (right) Implied timescale analysis for MSM construction. Solid lines indicate the maximum likelihood MSM estimate, while the dashed lines show the mean implied timescale bounded by a 95% confidence interval. A lag time of 5 ps was selected.

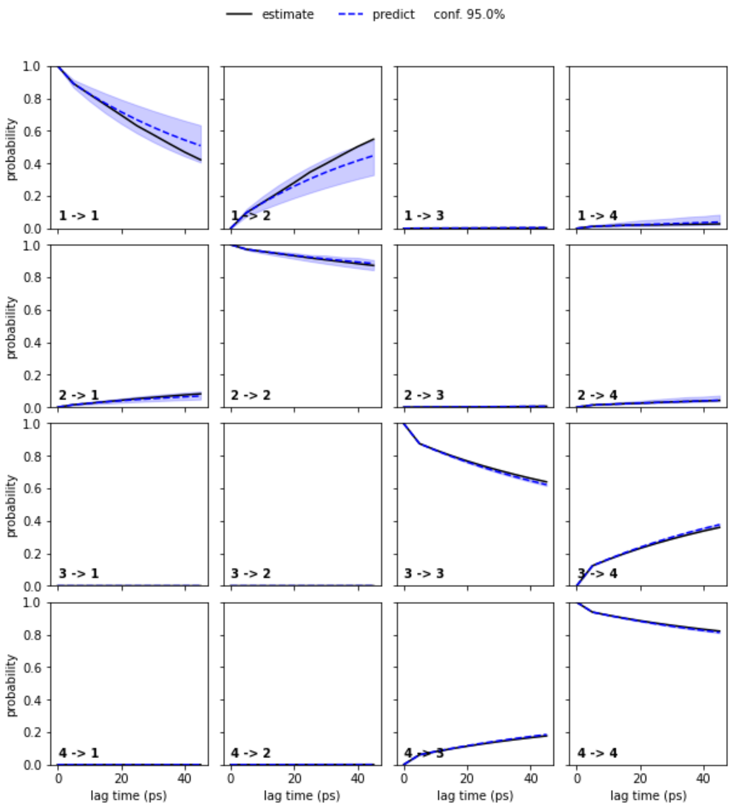

Figure S16: (Chapman-Kolmogorov (CK) test using 4 macrostates. Black lines indicate estimates, while the dashed blue lines are the predictions bounded by a 95% confidence interval)

ADP reference atomistic

For the generalization set we perform state space clustering using 100 kmeans centers first over the reference atomistic dataset which is taken from Ref.[14] These same 100 clusters then define state assignments for MSMs built on both the backmapped CG reconstructed data and the original CGSchNet[11] simulation.

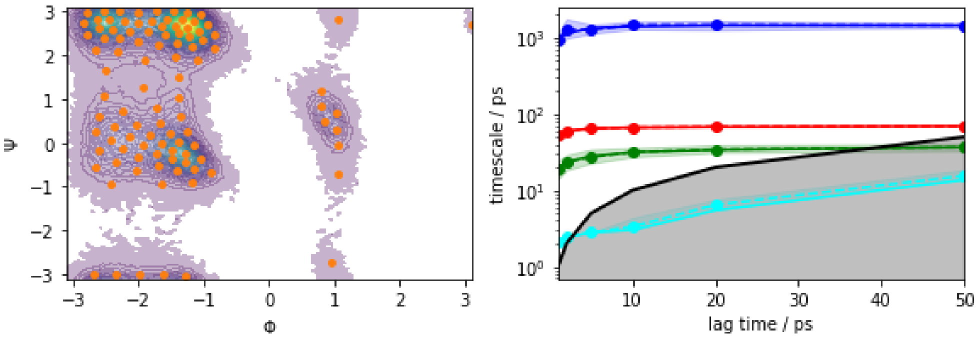

Figure S17: (left) State space clustering using 100 k-means centers fit to the reference atomistic data taken from Ref.[14] shown as orange dots over a contour of the data density. (right) Implied timescale analysis for MSM construction. Solid lines indicate the maximum likelihood MSM estimate, while the dashed lines show the mean implied timescale bounded by a 95% confidence interval. A lag time of 5 ps was selected.

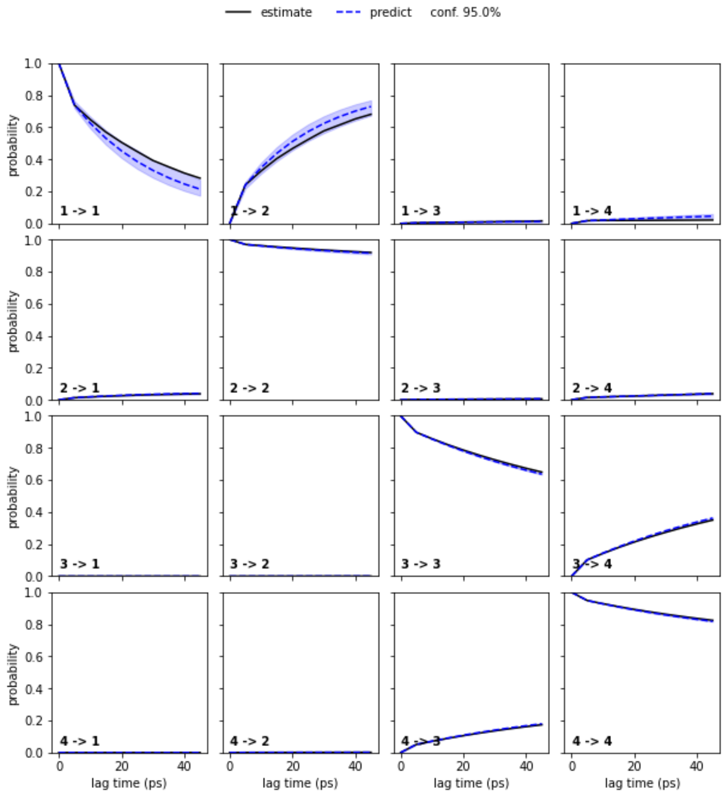

Figure S18: (Chapman-Kolmogorov (CK) test using 4 macrostates. Black lines indicate estimates, while the dashed blue lines are the predictions bounded by a 95% confidence interval)

ADP generalization set reconstructed

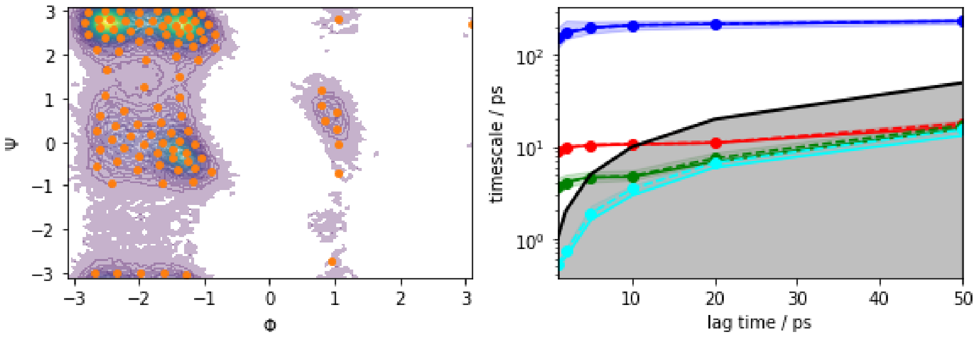

Figure S19: (left) State space clustering of the backmapped CGSchNet data using 100 k-means centers taken from the reference atomistic data shown as orange dots over a contour of the data density. (right) Implied timescale analysis for MSM construction. Solid lines indicate the maximum likelihood MSM estimate, while the dashed lines show the mean implied timescale bounded by a 95% confidence interval. A lag time of 5 ps was selected.

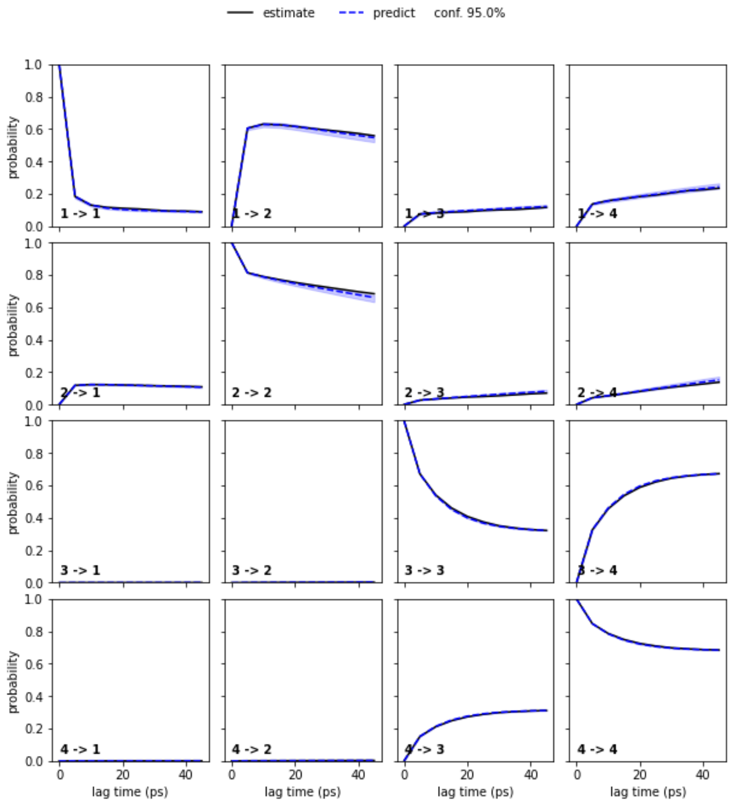

Figure S20: (Chapman-Kolmogorov (CK) test using 4 macrostates. Black lines indicate estimates, while the dashed blue lines are the predictions bounded by a 95% confidence interval)

ADP CGSchNet simulation

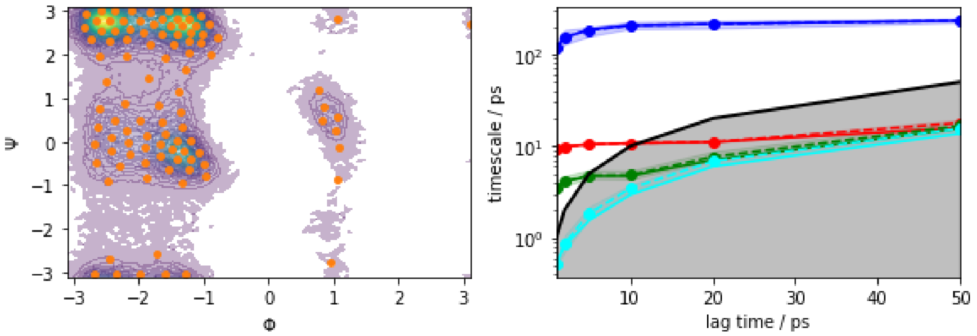

Figure S21: (left) State space clustering of the original coarse grained CGSchNet data using 100 k-means centers taken from the reference atomistic data shown as orange dots over a contour of the data density. (right) Implied timescale analysis for MSM construction. Solid lines indicate the maximum likelihood MSM estimate, while the dashed lines show the mean implied timescale bounded by a 95% confidence interval. A lag time of 5 ps was selected.

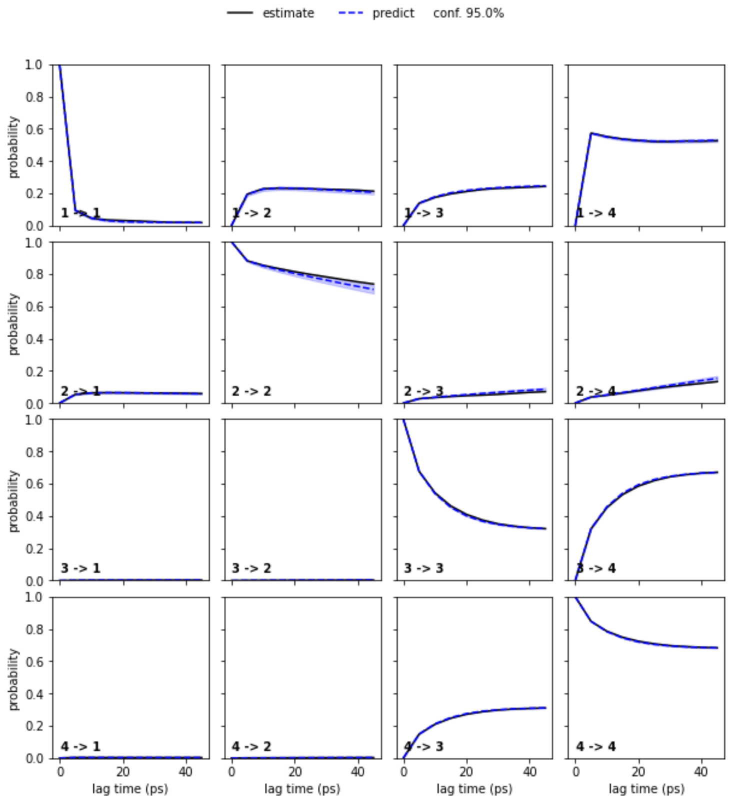

Figure S22: (Chapman-Kolmogorov (CK) test using 4 macrostates. Black lines indicate estimates, while the dashed blue lines are the predictions bounded by a 95% confidence interval)

1.5 Chignolin

Energetics

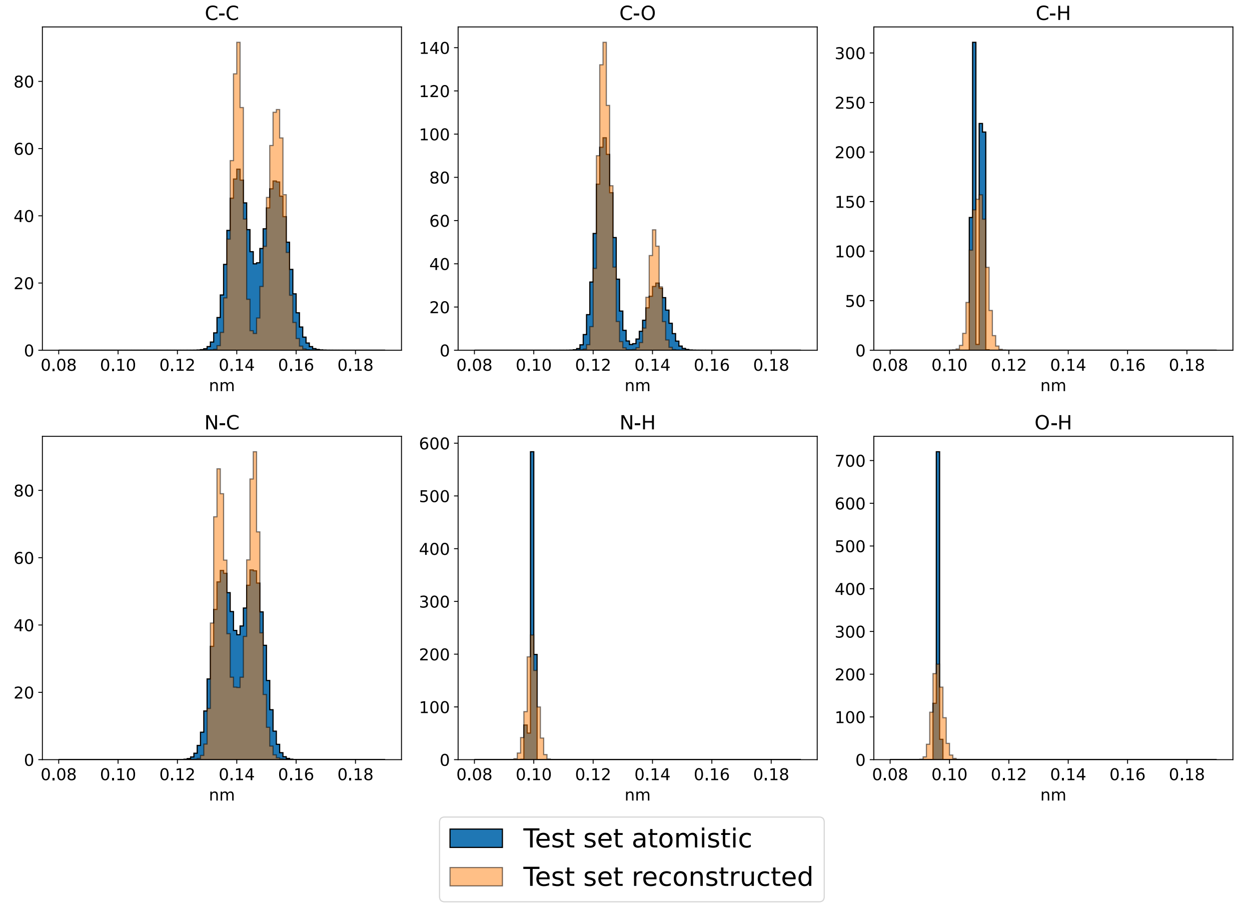

Figure S23: Bond length distributions delineated by element participation for the in distribution test set atomistic and backmapped trajectories for CLN.

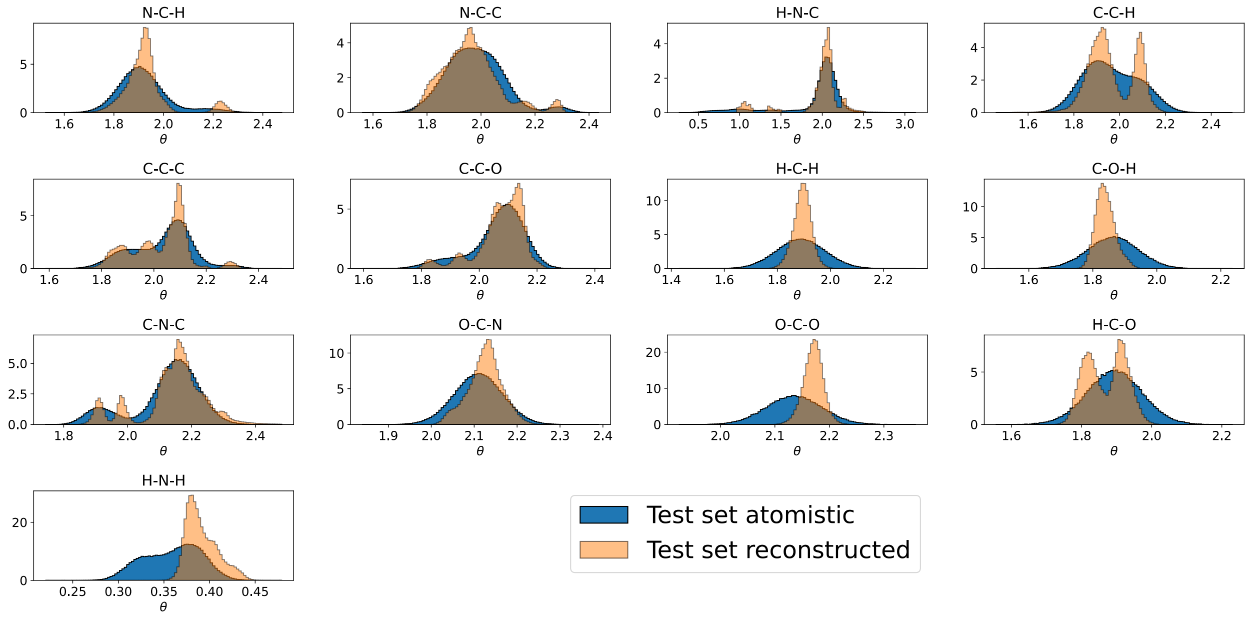

Figure S24: Atomic angle distributions delineated by element participation for the in distribution test set atomistic and backmapped trajectories for CLN.

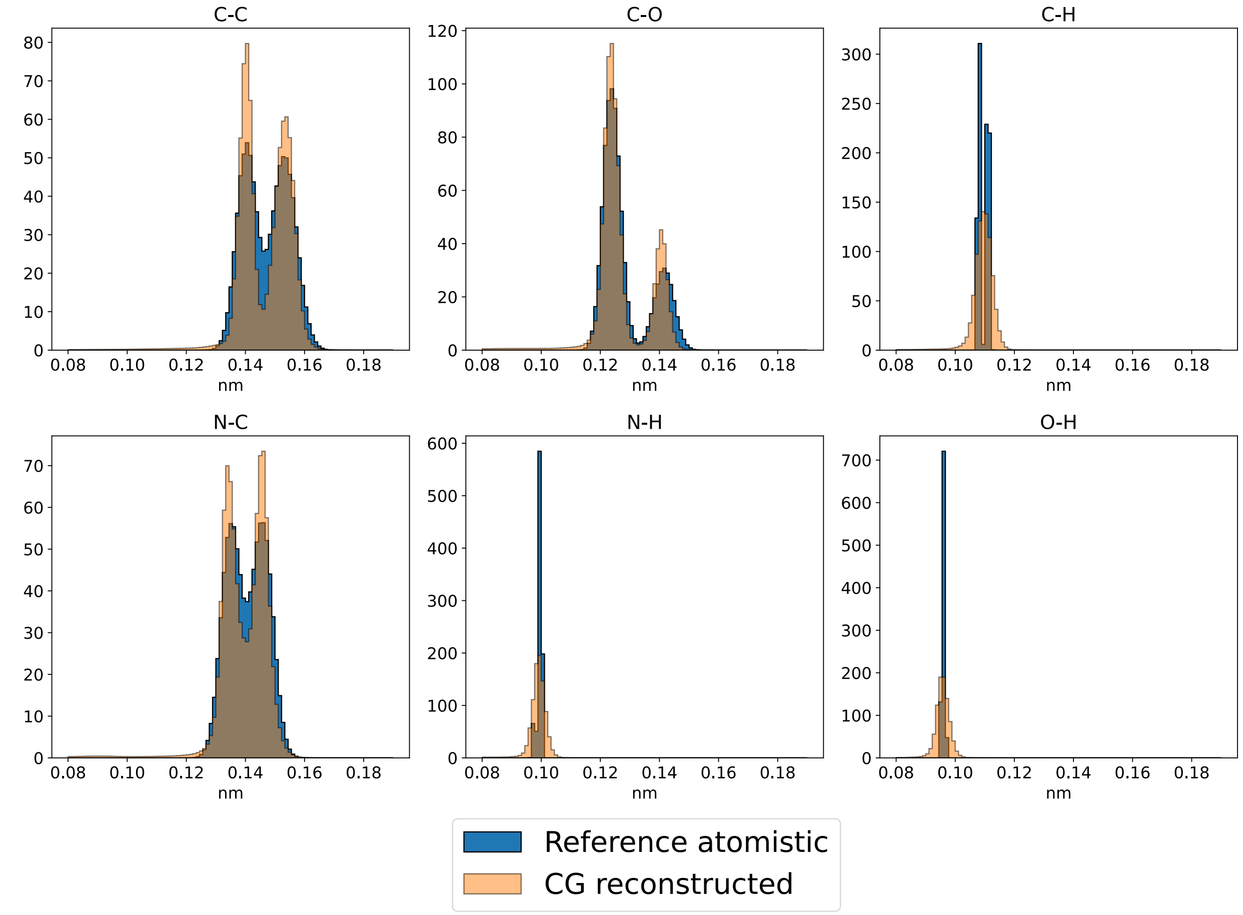

Figure S25: Bond length distributions delineated by element participation for the generalization test reference atomistic and backmapped CGSchNet trajectories for CLN.

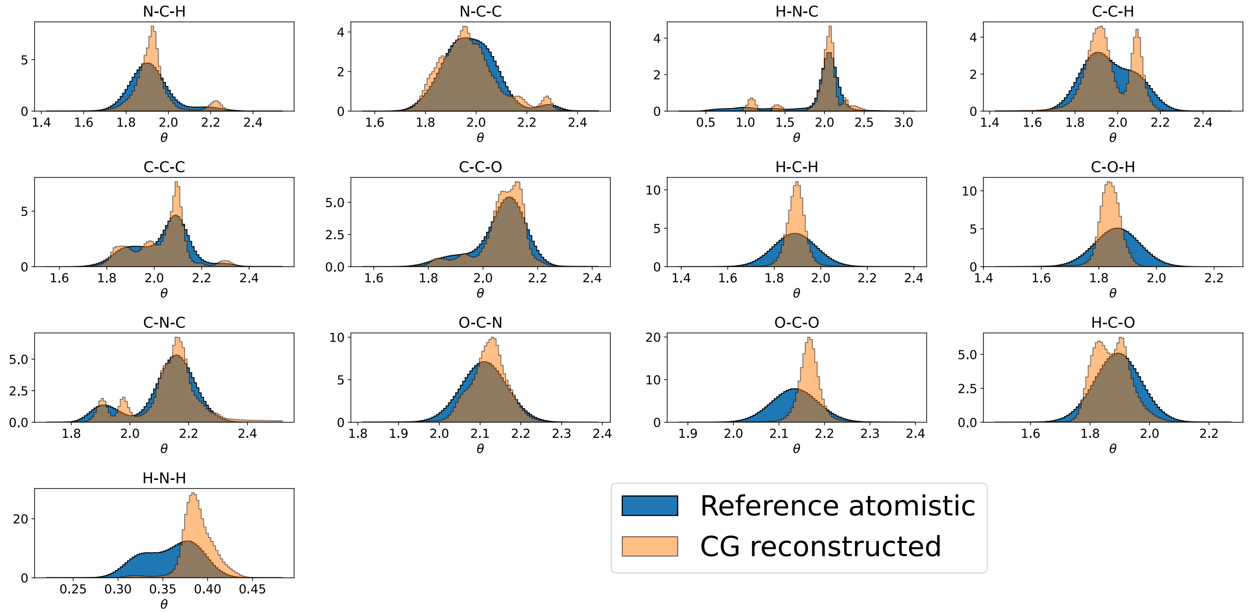

Figure S26: Atomic angle distributions delineated by element participation for the generalization test reference atomistic and backmapped CGSchNet trajectories for CLN.

Thermodynamics

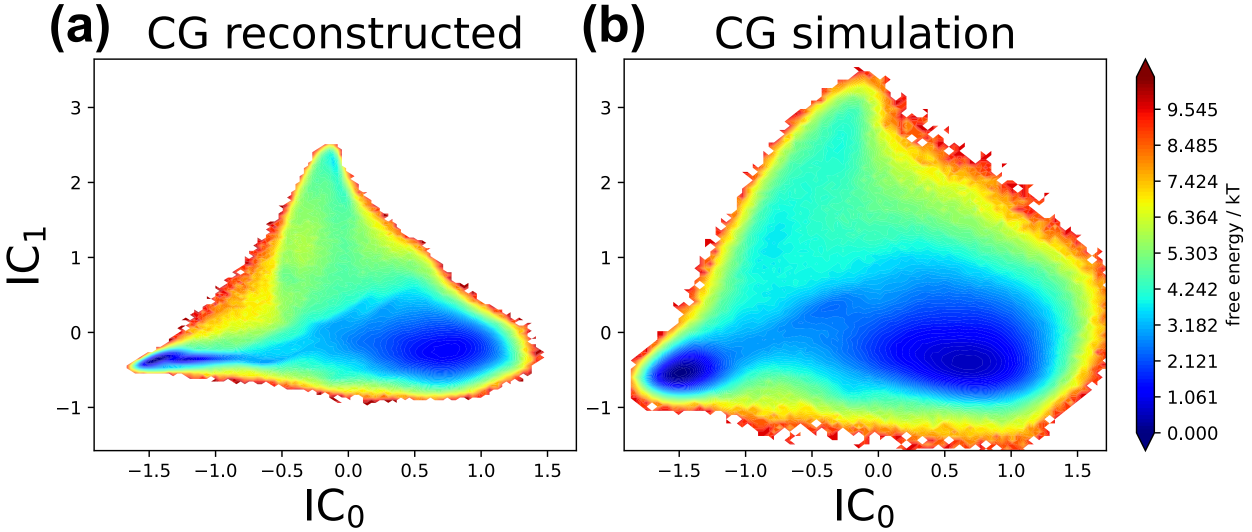

Figure S27: Comparison of MSM-reweighed FES for CLLN along the first two Independent Components (ICs) of a TICA model fit to the reference atomistic dataset between the **(a)** the backmapped CG reconstructed trajectory and **(b)** the original CG simulation performed with CGSchNet.

Kinetics

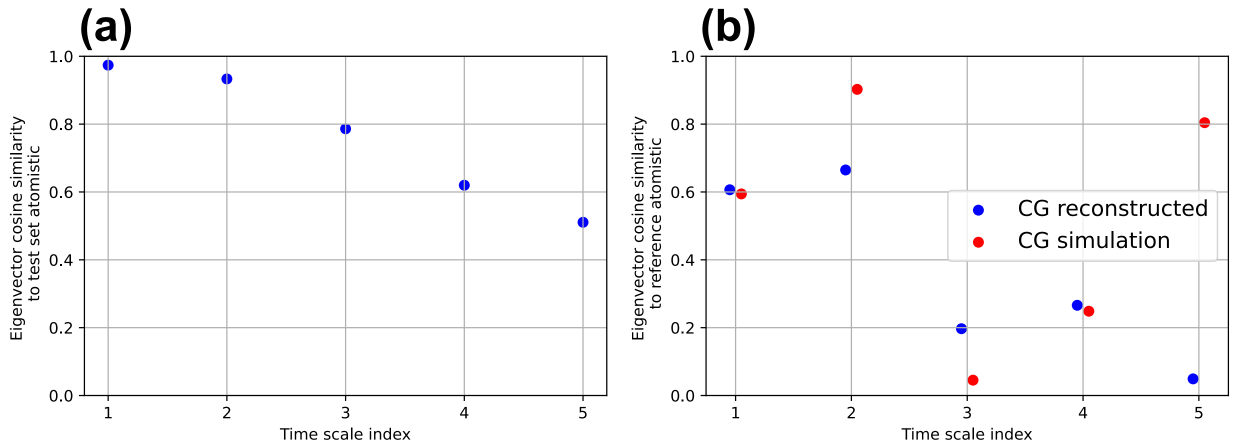

Figure S28: Cosine similarity between recovered MSM eigenvectors of atomistic and backmapped CLN trajectories. **(a)** The in distribution test set cosine similarity between atomistic and backmapped trajectories. **(b)** Cosine similarity between MSM eigenvectors of the backmapped data and the original CG data with respect to the reference atomistic data.

CLN MSM construction and validation

We construct Markov State Models (MSMs) to facilitate the comparison of the kinetics between the reference atomistic data and our backmapped trajectories in Sec. 3.2.3.

To construct MSMs for CLN we first featurize the reference atomistic data taken from Ref.[15] using the 45 pairwise α-carbon distances between each pair of amino acid residues. We then perform Time-lagged Independent Component Analysis (TICA) on this atomistic data using these pairwise distance features where we retain the first two Independent Components (ICs). We perform state decomposition in this TICA space using k-means clustering with 150 centroids. With the same residue contact featurization for other atomistic, backmapped or CG data we use our learned TICA model to project other data into a common TICA space. Independent MSMs are subsequently built for each separate dataset with state assignments generated in this common TICA space from the same previously identified 150 k-means centers in the reference atomistic dataset. In the case if not all 150 states are occupied MSMs are constructed only using the subset of occupied states for that data set. Operating within this shared TICA and clustering space allows direct comparison between the different MSM recovered timescales and processes. Associated state space clustering plots, implied timescale

analysis for lag time selection and Chapman-Kolmogorow (CK) tests for Markovianity for MSMs built on the different CLN datasets in this work are shown below.

CLN reference atomistic

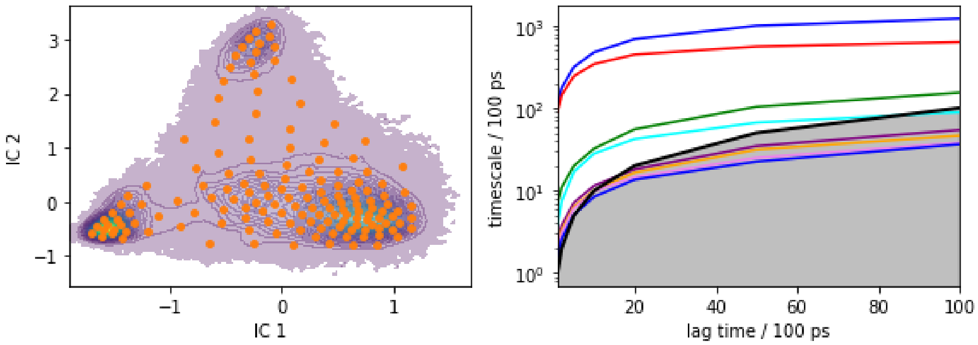

Figure S29: (left) Clustering in TICA space using 150 k-means centers fit to the reference atomistic dataset taken from Ref.[15] shown as orange dots over a contour of the data density. (right) Implied timescale analysis for MSM construction. Solid lines indicate the maximum likelihood MSM estimate. A lag time of 4 ns (40 frames) was selected.

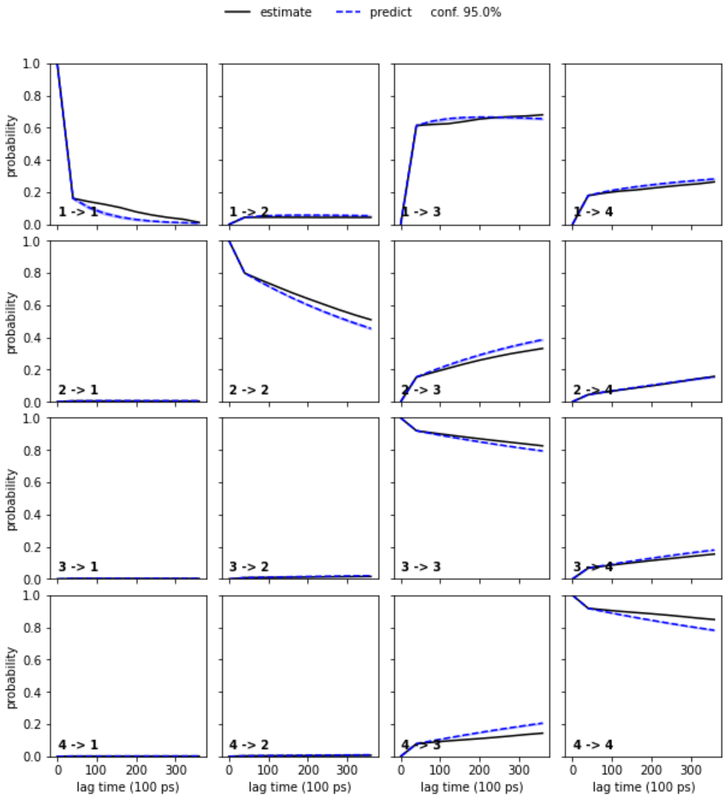

Figure S30: (Chapman-Kolmogorov (CK) test using 4 macrostates. Black lines indicate estimates while the dashed blue lines are the predictions bounded by a 95% confidence interval)

CLN in distribution test set atomistic

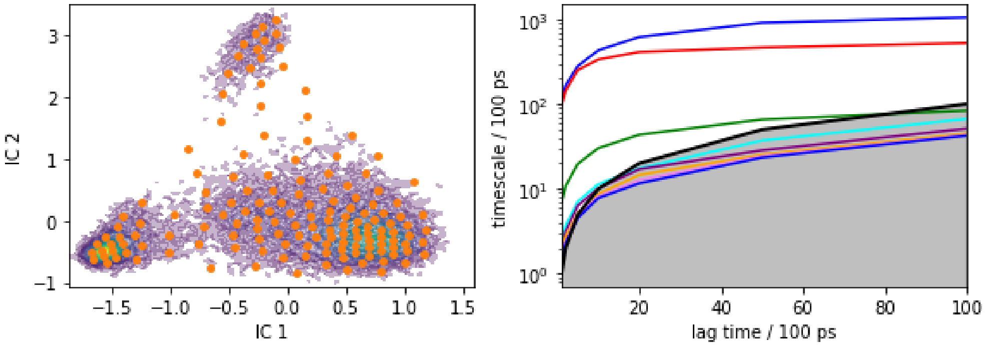

Figure S31: (left) State space clustering for the atomistic in distribution test set data using the same TICA projection and 150 k-means centers from the reference atomistic dataset shown as orange dots over a contour of the data density. (right) Implied timescale analysis for MSM construction. Solid lines indicate the maximum likelihood MSM estimate. A lag time of 4 ns (40 frames) was selected.

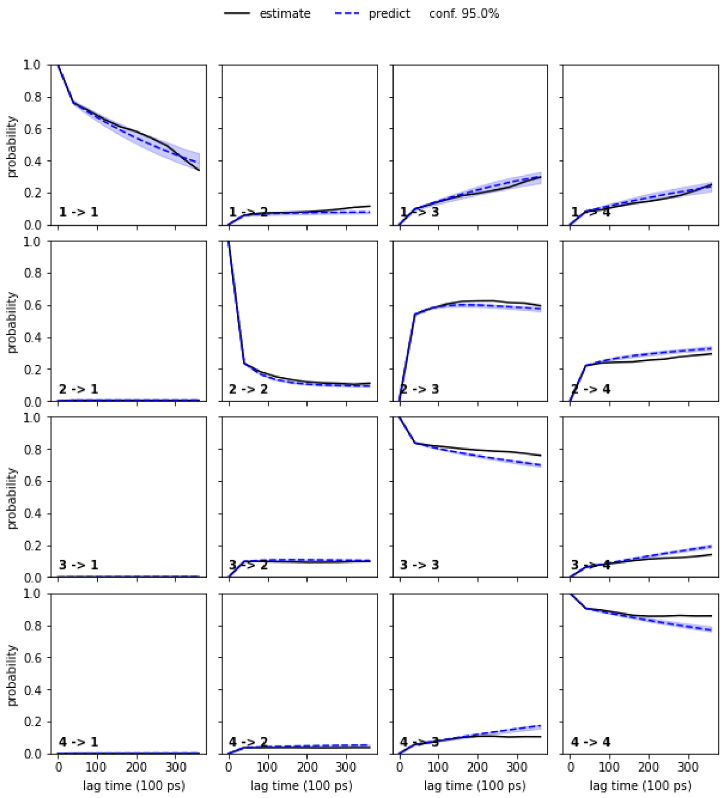

Figure S32: (Chapman-Kolmogorov (CK) test using 4 macrostates. Black lines indicate estimates while the dashed blue lines are the predictions bounded by a 95% confidence interval)

CLN in distribution test set reconstructed

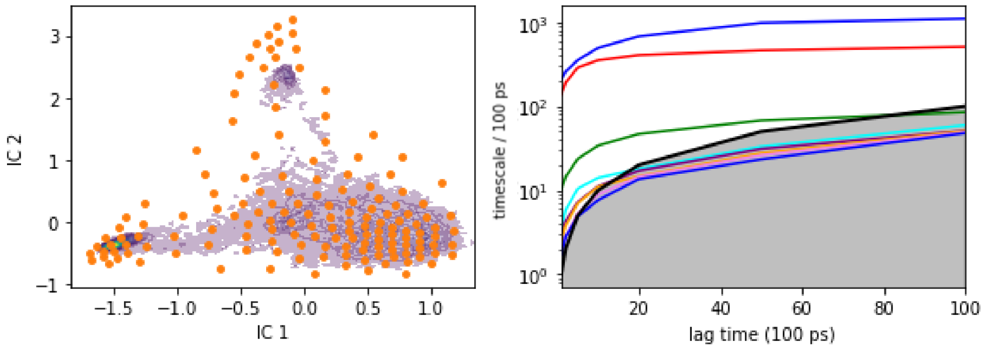

Figure S33: (left) State space clustering for the backmapped in distribution test set data using the same TICA projection and 150 k-means centers from to the reference atomistic dataset shown as orange dots over a contour of the data density. (right) Implied timescale analysis for MSM construction. Solid lines indicate the maximum likelihood MSM estimate. A lag time of 4 ns (40 frames) was selected.

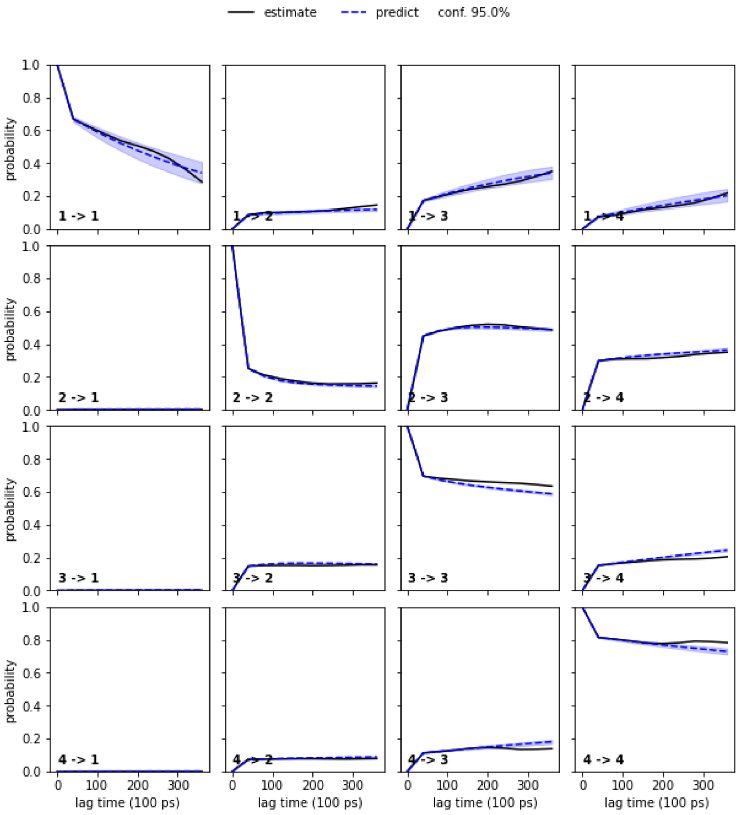

Figure S34: (Chapman-Kolmogorov (CK) test using 4 macrostates. Black lines indicate estimates while the dashed blue lines are the predictions bounded by a 95% confidence interval)

CLN generalization set reconstructed

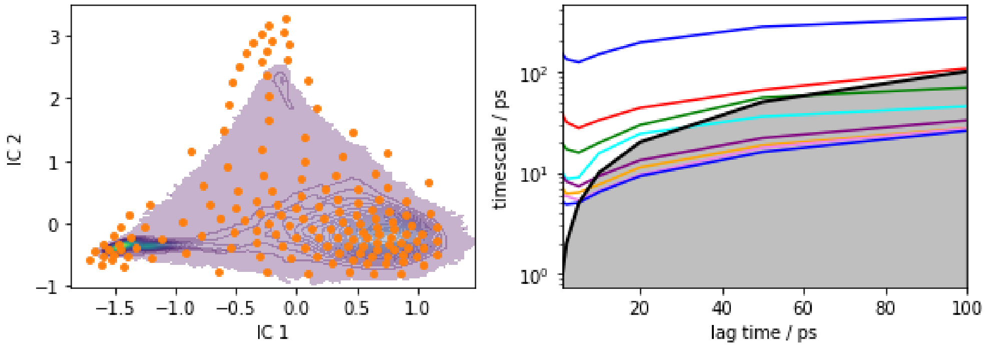

Figure S35: (left) State space clustering for the backmapped CGSchNet simulation data using the same TICA projection and 150 k-means centers from the reference atomistic dataset shown as orange dots over a contour of the data density. (right) Implied timescale analysis for MSM construction. Solid lines indicate the maximum likelihood MSM estimate. A lag time of 4 ns (40 frames) was selected.

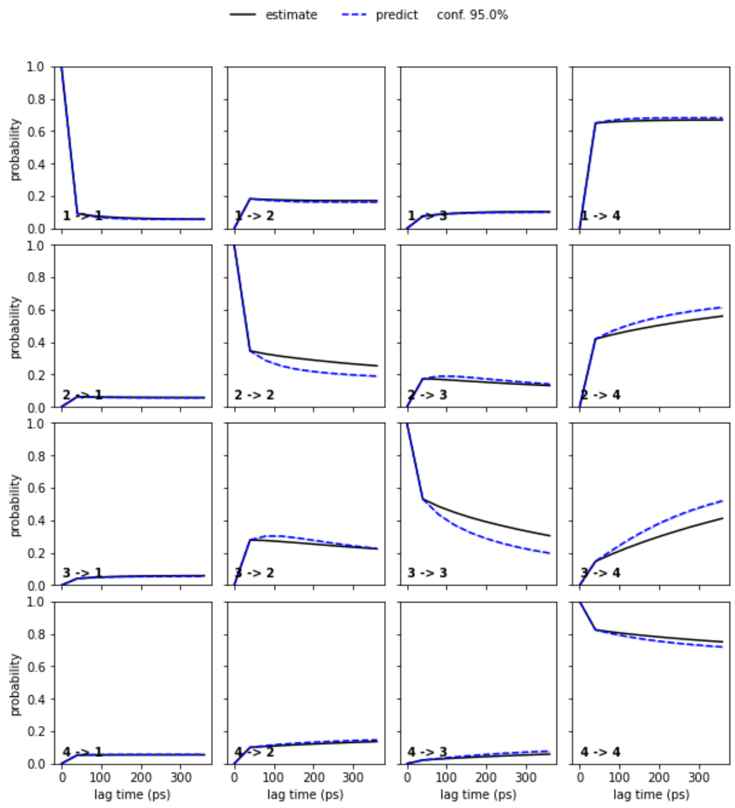

Figure S36: (Chapman-Kolmogorov (CK) test using 4 macrostates. Black lines indicate estimates while the dashed blue lines are the predictions bounded by a 95% confidence interval)

CLN CGSchNet simulation

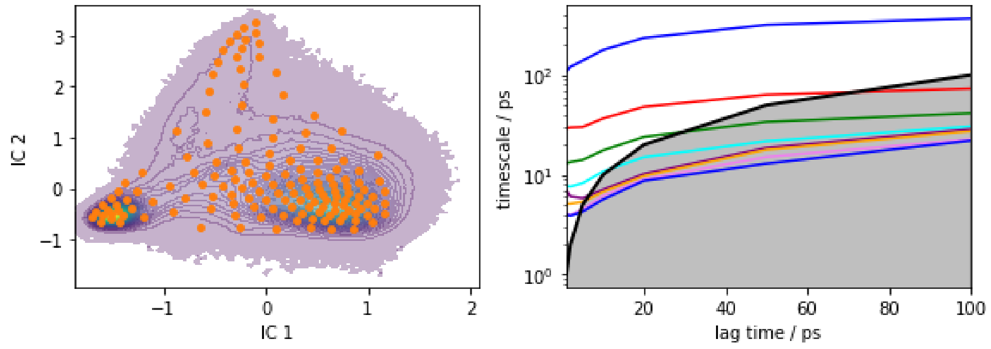

Figure S37: (left) State space clustering for the original CGSchNet simulation data using the same TICA projection and 150 k-means centers from the reference atomistic dataset shown as orange dots over a contour of the data density. (right) Implied timescale analysis for MSM construction. Solid lines indicate the maximum likelihood MSM estimate. A lag time of 4 ns (40 frames) was selected.

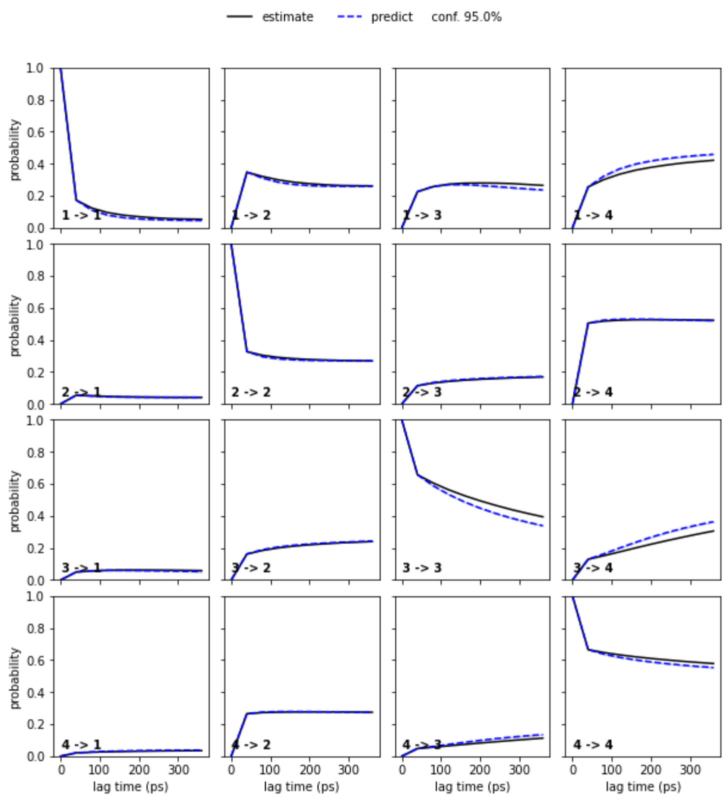

Figure S38: (Chapman-Kolmogorov (CK) test using 4 macrostates. Black lines indicate estimates while the dashed blue lines are the predictions bounded by a 95% confidence interval)

References

(1) Shmilovich, K.; Stieffenhofer, M.; Charron, N. E.; Hoffmann, M. Supporting data for "Temporally coherent backmapping of molecular trajectories from coarse-grain to atomistic resolution". 2022; `https://petreldata.net/mdf/detail/shmilovich_supporting_temporally_resolution_v1.6`, (accessed May 11, 2022).

(2) Eastman, P.; Swails, J.; Chodera, J. D.; McGibbon, R. T.; Zhao, Y.; Beauchamp, K. A.; Wang, L.-P.; Simmonett, A. C.; Harrigan, M. P.; Stern, C. D., et al. OpenMM 7: Rapid development of high performance algorithms for molecular dynamics. *PLoS Comput. Biol.* **2017**, *13*, e1005659.

(3) Lindorff-Larsen, K.; Piana, S.; Palmo, K.; Maragakis, P.; Klepeis, J. L.; Dror, R. O.; Shaw, D. E. Improved side-chain torsion potentials for the Amber ff99SB protein force field. *Proteins: Struct., Funct., Bioinf.* **2010**, *78*, 1950–1958.

(4) Brooks, B. R.; Brooks III, C. L.; Mackerell Jr, A. D.; Nilsson, L.; Petrella, R. J.; Roux, B.; Won, Y.; Archontis, G.; Bartels, C.; Boresch, S., et al. CHARMM: the biomolecular simulation program. *J. Comput. Chem.* **2009**, *30*, 1545–1614.

(5) Lee, J.; Cheng, X.; Swails, J. M.; Yeom, M. S.; Eastman, P. K.; Lemkul, J. A.; Wei, S.; Buckner, J.; Jeong, J. C.; Qi, Y., et al. CHARMM-GUI input generator for NAMD, GROMACS, AMBER, OpenMM, and CHARMM/OpenMM simulations using the CHARMM36 additive force field. *J. Chem. Theory Comput.* **2016**, *12*, 405–413.

(6) Jo, S.; Kim, T.; Iyer, V. G.; Im, W. CHARMM-GUI: a web-based graphical user interface for CHARMM. *J. Comput. Chem.* **2008**, *29*, 1859–1865.

(7) Fu, H.; Li, C.; Liu, X.; Gao, J.; Celikyilmaz, A.; Carin, L. Cyclical annealing schedule: A simple approach to mitigating kl vanishing. *arXiv preprint arXiv:1903.10145* **2019**, Submission date: 25 Mar 2019, DOI:10.48550/arXiv.1903.10145.

(8) Falcon, W., et al. Pytorch lightning. *GitHub. Note: https://github.com/PyTorchLightning/pytorch-lightning* **2019**, *3*, 6.

(9) Doerr, S.; Majewski, M.; Pérez, A.; Kramer, A.; Clementi, C.; Noe, F.; Giorgino, T.; De Fabritiis, G. Torchmd: A deep learning framework for molecular simulations. *J. Chem. Theory Comput.* **2021**, *17*, 2355–2363.

(10) Wang, W.; Gómez-Bombarelli, R. Coarse-graining auto-encoders for molecular dynamics. *npj Comput. Mater.* **2019**, *5*, 1–9.

(11) Husic, B. E.; Charron, N. E.; Lemm, D.; Wang, J.; Pérez, A.; Majewski, M.; Krämer, A.; Chen, Y.; Olsson, S.; de Fabritiis, G., et al. Coarse graining molecular dynamics with graph neural networks. *J. Chem. Phys.* **2020**, *153*, 194101.

(12) Chen, Y.; Krämer, A.; Charron, N. E.; Husic, B. E.; Clementi, C.; Noé, F. Machine learning implicit solvation for molecular dynamics. *J. Chem. Phys.* **2021**, *155*, 084101.

(13) Yi, Z.; Lindner, B.; Prinz, J.-H.; Noé, F.; Smith, J. C. Dynamic neutron scattering from conformational dynamics. II. Application using molecular dynamics simulation and Markov modeling. *J. Chem. Phys.* **2013**, *139*, 11B601_1.

(14) Wehmeyer, C.; Noé, F. Time-lagged autoencoders: Deep learning of slow collective variables for molecular kinetics. *J. Chem. Phys.* **2018**, *148*, 241703.

(15) Wang, J.; Olsson, S.; Wehmeyer, C.; Pérez, A.; Charron, N. E.; De Fabritiis, G.; Noé, F.; Clementi, C. Machine learning of coarse-grained molecular dynamics force fields. *ACS Cent. Sci.* **2019**, *5*, 755–767.
